# Mechanically Durable Intrinsically Stretchable Neuromorphic Devices via Molecular Microstructure Design

**DOI:** 10.1002/smll.202512071

**Published:** 2026-07-10

**Authors:** Kwan‐Nyeong Kim, Ho‐Eon Baek, Min‐Jun Choi, Min‐Jun Sung, Seung‐Woo Lee, Chae‐Yun Song, Hyun‐Haeng Lee, Sangjun Ma, Karina Ayu Larasati, Jeong‐Yun Sun, Hea‐Lim Park, Yun‐Hi Kim, Tae‐Woo Lee

**Affiliations:** ^1^ Department of Materials Science and Engineering Seoul National University Seoul Republic of Korea; ^2^ Department of Chemistry and RIMA Gyeongsang National University Jinju Republic of Korea; ^3^ Department of Materials Science and Engineering Seoul National University of Science and Technology Seoul Republic of Korea; ^4^ Research Institute of Advanced Materials Seoul National University Seoul Republic of Korea; ^5^ Department of Materials Science and Engineering Interdisciplinary Program in Bioengineering Institute of Engineering Research Soft Foundry Seoul National University Seoul Republic of Korea; ^6^ SN Display Co., Ltd. Seoul Republic of Korea

**Keywords:** neuromorphic electronics, organic semiconductors, stretchable electronics

## Abstract

Intrinsically stretchable neuromorphic devices (ISNDs) have been widely investigated for intelligent wearable on‐device computing. However, conventional material design strategies that soften the polymer conjugated moiety to impart stretchability have shown limited mechanical durability, typically 10^3^ cycles at 50% strain, with severe electrical degradation. Here, we present a highly durable ISND that maintains stable electrical performance for up to 10^5^ cycles at 50% strain, enabled by molecularly controlling chain stacking of the semiconducting polymer. This is achieved by incorporating a microstructure‐controlling moiety into the polymer backbone, which modulates the chain packing from a bundle‐like to a mesh‐like structure. The resulting mesh‐like morphology forms robust and long‐range percolation networks that preserve charge transport pathways and structural integrity under mechanical deformation. Utilizing this material, we fabricate ISNDs that exhibit device‐level stretchability of up to 150% and exceptional cyclic stability, with less than 15% variation in output current after 10^5^ cycles at 50% strain. Furthermore, we demonstrate reliable on‐device artificial intelligence using reservoir computing, with consistent classification accuracy maintained even after 10^5^ mechanical cycling at 50% strain. This work offers a molecular design strategy for tuning semiconductor film morphology, achieving mechanical reliability in stretchable neuromorphic electronics for future wearable and biomedical systems.

## Graphical abstract

This study reports intrinsically stretchable neuromorphic devices that exhibit outstanding mechanical stability while fully preserving their electrical characteristics over 10^5^ repeated deformation cycles under 50% tensile strain. Such robustness is achieved through a rational molecular design strategy that systematically modulates the chain packing of semiconducting polymers from a tightly packed bundle‐like microstructure to a mechanically compliant mesh‐like microstructure.

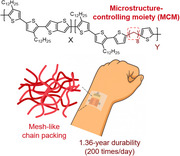

## 1 Introduction

Wearable neuromorphic devices for artificial intelligence have recently emerged as a central focus in next‐generation on‐body sensory processing. Such applications demand electronic platforms that can directly handle and interpret bio‐signals at or near the acquisition site, such as human skin and organs, thereby minimizing latency and reducing reliance on remote data transmission through rigid computing units [1, 2]. To achieve this, these devices should operate reliably under inevitable mechanical challenges such as stretching, bending, and twisting that repeatedly occur during daily body motions, requiring stretchability and mechanical durability. Accordingly, achieving high levels of stretchability and mechanical durability is of paramount importance.

To address these requirements, intrinsically stretchable neuromorphic devices (ISNDs) using semiconducting polymers (SPs) have been proposed as a promising class of devices, uniquely combining skin‐like mechanical compliance with neuro‐inspired signal processing capabilities [3, 4, 5]. By emulating the functional properties of biological synapses, ISNDs offer localized signal processing and memory functions with enhanced energy efficiency, compactness, and user comfort, making them highly attractive for on‐body artificial intelligence applications [6, 7, 8]. To enable seamless integration on human skin and consistent neuromorphic performance, ISNDs should maintain stable electrical characteristics under repetitive mechanical deformation. A key design consideration is the preservation of continuous charge transport pathways within the SP channel layers without generating any cracks under mechanical deformation and after repetitive stretching, which requires precise microstructural control to mitigate mechanical disruptions under strain [9].

To impart intrinsic stretchability to SPs, early strategies primarily relied on blending SPs with soft elastomers, forming physically mixed systems that introduce mechanical compliance [10, 11, 12, 13, 14, 15, 16, 17, 18, 19]. While such polymer‐elastomer blends have demonstrated static stretchability up to 100%, their mechanical stability rapidly deteriorates under cyclic deformation, with strain tolerance typically up to ∼30%, significantly below the 40% threshold required for reliable operation on human skin, such as the forearm [20]. To overcome this limitation, subsequent approaches have focused on synthesizing intrinsically stretchable SPs by introducing dynamic moieties directly into the conjugated backbone [7, 21]. Although these molecular design strategies have improved stretchability, achieving 50% strain tolerance over ∼10^3^ cycles, they still suffer from substantial electrical degradation (∼47% loss in output current). Moreover, in terms of their mechanical durability, they fall far short of the minimum endurance requirements for industrial relevance (e.g., folding endurance of ∼10^5^ cycles, as demanded by commercial devices such as the Galaxy Fold series [22]). Therefore, the development of a new class of stretchable SPs that can simultaneously provide high stretchability and mechanical durability remains a critical challenge.

**FIGURE 1:**
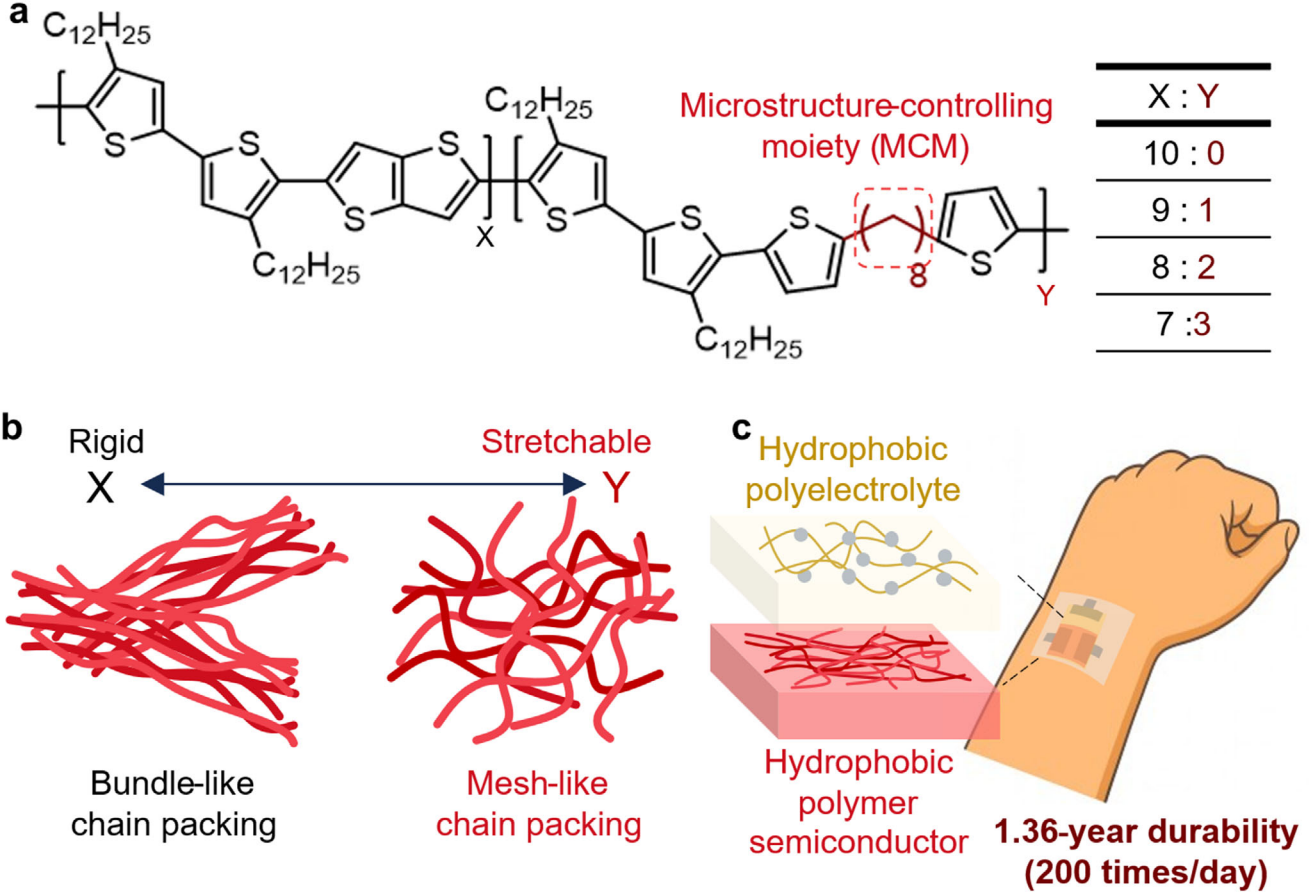
Microstructure‐controlling semiconducting polymer design for ISNDs. (a) Chemical structure of the semiconducting polymer with tunable X:Y ratios, where a microstructure‐controlling moiety (MCM) is introduced to modulate polymer microstructure. Four compositions (X:Y = 10:0, 9:1, 8:2, and 7:3) are used to systematically investigate morphology and mechanical properties. (b) Schematic representation of molecular packing transition from rigid bundle‐like structures (X‐rich) to stretchable mesh‐like configuration (Y‐rich) induced by controlling microstructure. (c) Device concept illustrating the integration of a hydrophobic semiconducting polymer layer with a hydrophobic polyelectrolyte for stable operation under mechanical strain. The ISND demonstrates excellent mechanical durability (∼10^5^ cycles at 50% strain), corresponding to ∼1.36 years of operation under daily 200‐cycle deformation.

In this study, to develop intrinsically stretchable and mechanically robust neuromorphic devices, we introduce a molecular design strategy that preserves the rigidity of the conjugated backbone while imparting mechanical compliance through an MCM‐mediated reconfiguration of polymer chain packing and film morphology. Specifically, instead of softening the conjugated moiety, we retain a rigid semiconducting backbone to ensure structural and electronic stability, while incorporating an MCM that enables microstructural tunability. By systematically optimizing the X:Y ratio between the conjugated moiety (X) and MCM segments (Y) (Figure 1a), the polymer morphology evolves from bundle‐like to mesh‐like percolated networks, enabling structurally controlled stretchability while maintaining the rigidity of the conjugated moiety (Figure 1b). This approach successfully identifies an optimal compositional window where high mechanical stretchability and high cyclic durability are simultaneously achieved. ISNDs fabricated from this material exhibit exceptional mechanical resilience, withstanding 10^5^ cycles at 50% strain, with less than 15% variation in output current, corresponding to over 1.36 years of daily use (200 cycles/day) (Figure 1c). Moreover, the devices retain stable neuromorphic performance under strain, achieving well‐preserved classification accuracies of ∼89% and ∼90% with negligible variation for handwritten and spoken digit recognition, respectively, via a reservoir computing framework. Collectively, this work establishes a new molecular design paradigm for intrinsically stretchable SPs that preserves backbone rigidity while incorporating an MCM to enhance both intrinsic stretchability and mechanical durability, thereby providing a viable pathway toward highly durable and high‐performance wearable neuromorphic systems.

## 2 Controlling Polymer Microstructure and Mechanical Stretchability

We synthesized poly[2,5‐bis(3‐tetradecylthiophen‐2‐yl)thieno[3,2‐b]thiophene] (PBTTT) derivatives incorporating thiophene–alkyl–thiophene as the MCM (Figure 1). The synthetic scheme, synthesis of polymers, and characterization of polymers are described in the (Note S1 and Figures S1–S23). During the film formation process, highly planar PBTTT chains inherently assemble into densely packed bundle‐like microstructures owing to strong interchain interactions [23]. Polyethylene segments adopt random‐coil conformations in the amorphous domain [24]. Therefore, by incorporating the alkyl chains as MCM unit into the planar PBTTT backbone, we are able to release the well‐defined rigid bundle packing of PBTTT, characteristic of strong *π*–*π* stacking, and reconfigure the morphology into a mesh‐like, loosely packed network (i.e., disordered network) due to disrupted molecular packing (Figure 1b). This controlled structural transition facilitates the development of long‐range percolation networks of conjugated moiety, thereby providing charge transport pathways while simultaneously imparting enhanced stretchability and mechanical compliance.

**FIGURE 2:**
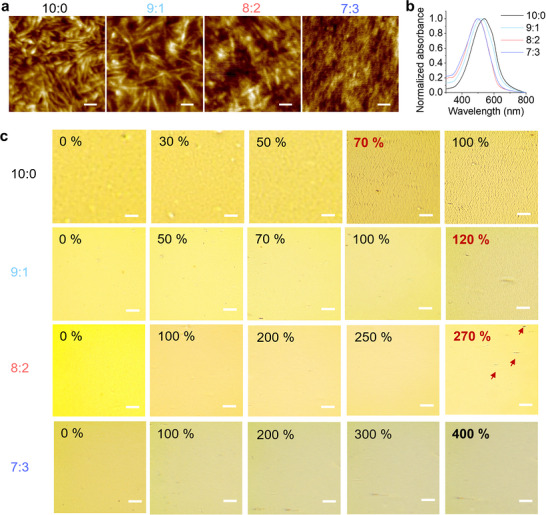
Controlling polymer morphology and stretchability. (a) Atomic force microscopy (AFM) images with varying X:Y ratios (10:0, 9:1, 8:2, and 7:3). The 10:0 sample exhibits highly ordered, fibrillar domains, while increasing Y content disrupts this order, resulting in finer and more disordered surface morphologies. These correspond to bundle‐like and mesh‐like chain packings, respectively. Scale bar: 500 nm. (b) UV–vis absorbance spectra of polymer films with varying X:Y ratios (10:0, 9:1, 8:2, and 7:3), showing gradual blue shifts and saturation of peak wavelengths, indicative of changes in molecular packing with increasing Y content. (c) Optical microscopy images of the films under increasing tensile strain. Crack initiation strain increases with Y content: the 10:0 and 9:1 film exhibit visible cracks at 70% and 120% strain, respectively, across the entire film area. The 8:2 film withstands up to 270% strain with localized cracking (red arrows), while the 7:3 film remains intact even at 400% strain, demonstrating superior mechanical stretchability. Scale bar: 10 µm.

To directly visualize the morphological evolution induced by incorporating MCM, we measured atomic force microscopy (AFM) images of polymer films with increasing Y content, ranging from 10:0 to 7:3 (Figure 2a). As the MCM ratio increases, these rigid stacked domains progressively fragment into smaller and less interconnected structures up to the 8:2 composition. Upon further increasing the MCM content to 7:3, fibrillar aggregation is largely suppressed, resulting in a uniform mesh‐like disordered network. This morphological transition and reduced molecular packing are quantitatively supported by the monotonic decrease in surface roughness from 2.399 nm (10:0) to 1.590 nm (9:1) and 1.178 nm (8:2), followed by a dramatic drop to 0.359 nm at 7:3 [25]. This abrupt reduction indicates that the transition from 8:2 to 7:3 represents a threshold beyond which bundle‐like strong aggregation is effectively disrupted (Figure S24). Consistent with the height analysis, AFM phase images further confirm the loss of rigid crystalline contrast and the emergence of a more homogeneous microstructure at the 7:3 composition (Figure S25). Such microstructural reconfiguration suggests that the disruption of rigid bundle packing promotes the formation of interconnected percolation networks, which can more effectively distribute mechanical strain while maintaining continuous charge transport pathways. This structural evolution underpins the enhanced mechanical compliance without compromising electronic functionality under tensile deformation.

Raman spectroscopy reveals a systematic evolution of the PBTTT backbone microstructure with increasing content of the microstructure‐controlling moiety (MCM) (Figure S26). The peak at 1391 cm^−^^1^ corresponds to the symmetric C─C stretching mode of the thiophene ring, while the peak at 1413 cm^−^^1^ originates from the symmetric C═C stretching vibration of the thienothiophene unit. The higher‐wavenumber peak at 1487–1489 cm^−^^1^ is assigned to the symmetric C═C stretching mode of the thiophene unit [26, 27]. Specifically, the C═C stretching mode of the thiophene unit exhibits a blue shift accompanied by peak broadening when the MCM is incorporated, which is a well‐established spectroscopic signature of increased backbone twisting and conformational disorder in PBTTT chains [26, 27]. In parallel, the increased intensity ratio of the thiophene C═C to C─C stretching modes in MCM‐incorporated samples, relative to pristine PBTTT, further indicates a progressive loss of local packing order induced by enhanced segmental flexibility [27]. Moreover, the relative intensity of the thiophene C═C stretching mode decreases compared to that of the more rigid thienothiophene unit, suggesting that MCM selectively enhances the conformational freedom of thiophene segments while preserving the rigidity of thienothiophene motifs. Notably, similar intensity ratios for 9:1 and 8:2 indicate that molecular packing is largely maintained up to 8:2, whereas the pronounced decrease observed for 7:3 implies a clear disruption of effective molecular packing beyond this threshold. This trend is consistent with AFM results, which show that the characteristic fibrillar morphology of PBTTT is preserved up to 8:2 but diminished in 7:3. Such selective softening introduces controlled microstructural disorder that facilitates chain rearrangement and stress dissipation under tensile deformation, thereby contributing to enhanced stretchability. As a result, the MCM‐induced microstructural disorder plays a critical role in enhancing the stretchability of PBTTT thin films without catastrophically disrupting charge‐transport pathways.

To further investigate the effect of microstructure controls on energetic states, we measured UV–vis absorption spectra of the polymer films with varying MCM content (Figure 2b). As the Y value in the backbone increased from 0 to 3, the main *π*–*π* ^*^ transition peak in absorbance spectra gradually shifted toward shorter wavelengths. This blue shift reflects a reduction in effective conjugation length and an increase in energetic disorder. This spectral evolution aligns with the observed morphological changes from a highly ordered bundle‐like packing (10:0, 9:1) to a more disordered, yet interconnected, mesh‐like configuration (8:2, 7:3). While this transition naturally reduces aggregation of polymer chains, it represents the intended microstructure reconfiguration strategy that relaxes packing constraints, thereby improving mechanical compliance and enabling enhanced stretchable functionality.

Grazing‐incident wide‐angle X‐ray scattering (GIWAXS) was performed to investigate the crystal orientation and packing evolution with increasing MCM content (Figure S27 and Table S1). GIWAXS analysis confirms that all compositions exhibit a dominant edge‐on molecular orientation, while the crystalline packing coherence systematically degrades as the MCM fraction increases. Quantitatively, the lamellar stacking distance gradually increases from 18.409 Å (10:0) to 19.090 Å (9:1), 19.091 Å (8:2), and further to 19.825 Å (7:3). In parallel, the π–π stacking distance increases monotonically from 4.306 Å (10:0) to 4.359 Å (9:1), 4.396 Å (8:2), and 4.473 Å (7:3), indicating progressive weakening of interchain interaction and crystallinity. In addition to the expanded packing distances, the (200) diffraction peak exhibits systematic broadening with increasing MCM content. The full width at half maximum increases, leading to a continuous reduction in the crystal coherence length (*L*_c_). Such progressive domain fragmentation weakens the mesoscale connectivity of crystalline regions and ultimately renders the percolation pathways for charge transport increasingly discontinuous. These results highlight that the controlled reduction of crystallinity with increasing MCM content is a critical design parameter for identifying the optimal balance between charge transport performance and stretchability.

Electrochemical properties were subsequently investigated using cyclic voltammetry to correlate the morphological transition with HOMO and LUMO energy levels (Figure S28 and Table S2). As the MCM content increased, the oxidation onset potential shifted toward more positive values and the reduction onsets toward more negative values, leading to a down‐shift (stabilization) of both HOMO (−5.03 to −5.12 eV) and LUMO (−2.84 to −2.90 eV) energy levels. The energetic stabilization is consistent with the microstructural evolution toward a mesh‐like morphology, where reduced *π*–*π* stacking limits electronic delocalization [28]. Importantly, this energetic modulation occurs concomitantly with the desired microstructure transformation, ensuring that the enhancement in mechanical stretchability is achieved without significantly altering the relative alignment of frontier orbitals.

We next conducted thermogravimetric analysis (TGA) to evaluate the thermal stability of the polymers with varying MCM content (Figure S29). All compositions exhibited 5% weight‐loss temperatures (*T*_d,5%_) exceeding 390°C, confirming the intrinsic thermal robustness. The 10:0 film showed the highest T_d,5%_ of 404°C, due to its densely packed bundle‐like morphology, which provides strong interchain interactions and enhanced resistance to thermal degradation. In MCM‐incorporated samples, a slight decrease in *T*_d,5%_ was observed (394°C for 9:1, 398°C for 8:2, and 390°C for 7:3), consistent with the microstructure transition toward a less ordered mesh‐like morphology. Despite this slight reduction, the consistently high *T*_d,5%_ across all compositions, indicates that incorporation of MCM maintains the intrinsic thermal robustness of the polymers, ensuring their suitability for reliable operation in stretchable neuromorphic devices.

To assess the effect of microstructure controls on mechanical properties, we conducted optical microscopy (OM) measurements of the polymer films under tensile strain on thermoplastic polyurethane (TPU) substrates, with compositions of 10:0, 9:1, 8:2, and 7:3 (Figure 2c). The 10:0 film exhibited visible crack formation at relatively low strain (70%), consistent with its rigid bundle‐like packing and limited ability to dissipate mechanical stress. In contrast, films with higher MCM ratios demonstrated significantly delayed crack initiation, with 120% and 270% crack‐onset thresholds for 9:1 and 8:2, respectively, and with the 7:3 composition sustaining up to 400% strain without observable fracture. This progressive enhancement in crack resistance directly correlates with the AFM images and UV–vis spectroscopy results, identifying bundle‐to‐mesh microstructural transition, wherein the mesh‐like morphology enables more effective stress redistribution and mechanical accommodation. These findings validate the effect of the MCM strategy in tuning polymer chain packing to enhance intrinsic stretchability, thereby meeting the demands of next‐generation wearable device applications.

## 3 Electrical and Synaptic Properties Tuned by MCM

The electrical characteristics of the PBTTT with varying MCM content were systematically evaluated using an electrolyte‐gated transistor with a solid‐state electrolyte gate dielectric to investigate the effect of microstructure reconfiguration on electrochemical doping.

**FIGURE 3:**
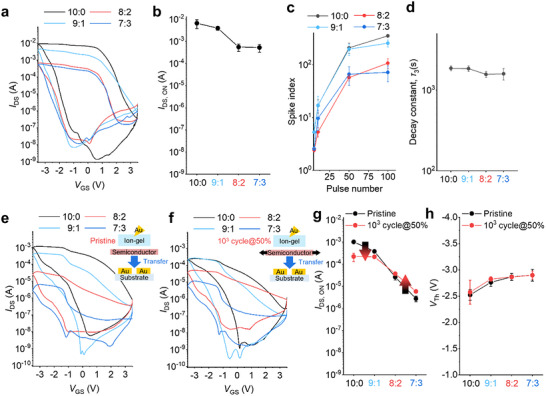
Electrical and synaptic characteristics tuned by MCM. (a) Transfer curves of ISNDs with varying X:Y (MCM) ratios (10:0, 9:1, 8:2, 7:3) plotted in absolute scale, showing systematic modulation of channel current with increasing MCM content. (b) *I*_DS,ON_ at *V*_GS_ = −3.5 V significantly decreases as MCM content increases, indicating a trade‐off between electrical performance and mechanical stretchability. (c) Spike index as a function of pulse number. ISNDs with higher MCM content show more gradual accumulation behavior, while 10:0 shows a steeper response. (d) *τ*_3_ extracted from spike decay curves, showing a decrease with increasing MCM content, indicating faster relaxation in more stretchable formulations. Collectively, increasing the MCM content gradually reduces *I*_DS_, spike response rate, and decay time, attributed to the loosening of chain packing from densely ordered to more relaxed structures. (e, f) Transfer curves of pristine (e) and mechanically cycled (10^3^ cycles at 50% strain) (f) semiconductor films. The results show that the mesh‐like chain packing formed in the 8:2 and 7:3: composition facilitates chain realignment along the charge transport direction between source and drain under deformation. (g) *I*_DS,ON_ values and relative changes after cyclic stretching. (h) *V*_Th_ values and relative changes after cyclic stretching.

Transfer curves were measured at a drain voltage (*V*_DS_) of −0.5 V, with gate voltage (*V*_GS_) swept from +3.5 V to −3.5 V (Figure 3a). We statistically evaluated transfer measurements for five devices fabricated on different substrates for each MCM content. The on‐current (*I*_DS,ON_) exhibited a reduction with increasing MCM ratio, coinciding precisely with the bundle‐to‐mesh morphological transition and reduction in chain packing confirmed by AFM images and absorbance spectra (Figure 3b). Threshold voltage (*V*_Th_) shifted negatively from −1.614 V (10:0) to −2.130 V (7:3) with increasing MCM content (Figure S30a), reflecting changes in the electrolyte/polymer interface due to reduced crystallinity. The increase in amorphous content is expected to raise the activation energy for electrochemical doping by weakening *π*–*π* electronic coupling. As a result, MCM‐incorporated devices require a higher gate bias to initiate bulk electrochemical doping and establish stable channel conduction, compared to pristine PBTTT‐based devices. Hysteresis voltage (*V*_Hysteresis_) slightly decreased with increasing MCM (Figure S30b). A narrower hysteresis window indicates faster dedoping during the reverse sweep in the more amorphous films, but the overall transfer characteristics, including hysteresis behavior, are well maintained across all compositions, confirming that the devices retain the memory functionality associated with reversible electrochemical doping. These results demonstrate that, although the bundle‐to‐mesh microstructural transition compromises the absolute current level, the essential transistor characteristics and memory behavior remain intact, thereby achieving the design objective of enhancing mechanical compliance without sacrificing core device functionality.

To assess neuromorphic functionality, we evaluated synaptic plasticity using pulsed‐gate measurements (Figure S31). All tests were conducted at *V*_DS_ = −0.5 V using gate pulses of −3.5 V amplitude, 100 ms width, and 100 ms intervals, with pulse counts of 5, 10, 50, and 100 to evaluate the spike number‐dependent plasticity (SNDP). The spike index at *n*th pulse calculated as the ratio of the current increment at the *n*th pulse to that at the first pulse, where each increment is obtained by subtracting the baseline current from the corresponding pulse current. The spike index at 100th pulse decreased from 350 (10:0) to 255 (9:1), 106 (8:2), and 71.6 (7:3) (Figure 3c), reflecting the influence of MCM content on synaptic response behavior. Similarly, the decay constant *τ*_3_ extracted via triexponential fitting from post‐stimulation current decay and indicative of long‐term memory behavior [29] decreased upon incorporating MCM  (Figure 3d; Figure S32). This trend is primarily attributed to a faster dedoping process in the more amorphous films with higher MCM content. While both the spike index and decay constant slightly decrease with increasing MCM, essential synaptic features, particularly potentiation, which constitutes the core functional plasticity required for efficient neuromorphic computing, such as reservoir computing, are well preserved even at the highest MCM content. This demonstrates that the designed bundle to mesh microstructural transition, while adjusting the amplitude and temporal characteristics of synaptic responses, retains the essential synaptic plasticity required for neuromorphic functionality. Furthermore, the controlled polymer morphology enables the seamless integration of enhanced mechanical compliance with stable synaptic behavior, offering a robust material platform for intrinsically stretchable neuromorphic devices.

In addition to the evaluation of SNDP, spike dynamics of the 8:2 composition, including spike‐voltage‐dependent plasticity (SVDP) and spike‐rate dependent plasticity (SRDP) were further measured (Figure S33). The pulse duration was fixed at 300 ms across all experiments to ensure consistent stimulation conditions. For SVDP, the gate voltage amplitudes were varied from −1.5 to −3.5 V. The PPF index exhibited a pronounced increase with higher spike amplitudes, from 163% at −1.5 V to 227% at −2.5 V and 256% at −3.5 V (Figure S33a,b). This trend highlights the capability of the device to modulate synaptic weight in proportion to the strength of presynaptic stimuli, emulating amplitude‐dependent facilitation observed in biological synapses. SRDP was evaluated by varying the inter‐spike interval from 100 to 3000 ms while maintaining a constant −3.5 V gate pulse (Figure S33c,d). The PPF index decreased monotonically with increasing intervals, ranging from 267% at 100 ms to 128% at 3000 ms. Such interval‐dependent decay reflects the expected decay in facilitation due to ionic relaxation within the polymer network, consistent with the temporal filtering behavior observed in natural synapses.

To assess the effect of MCM on charge transport behavior and mechanical stability, the films were subjected to 10^3^ cycles of 50% tensile strain, and their transfer characteristics were measured before and after deformation (Figure 3e,f). We statistically evaluated transfer measurements for five devices fabricated on different substrates for each MCM content. As confirmed by comprehensive microscopy and spectroscopy analyses, including AFM, GIWAXS, Raman spectroscopy, and UV–vis absorbance, the *I*_DS,ON_ at *V*_GS_ = −3.5 V gradually decreases with increasing MCM content due to reduced interchain electronic coupling and crystallinity. Devices using the 10:0 and 9:1 compositions exhibit a noticeable decrease in *I*_DS,ON_ after cyclic stretching, indicating that the rigid bundle‐like packing fails to preserve continuous charge‐transport pathways under repeated mechanical deformation (Figure 3g). In contrast, the 8:2 composition shows a clear increase in *I*_DS,ON_ after cyclic stretching, while the 7:3 composition exhibits an even larger increase (Figure 3g). Transconductance (*g*_m_) values follow the same trend (Figure S34). This behavior is attributed to the mesh‐like microstructure, which facilitates polymer chain realignment along the source–drain direction and preserves long‐range percolation pathways under strain owing to enhanced mechanical compliance and stretchability. In addition, the absolute threshold voltage |*V*_Th_| increases with the incorporation of MCM, while the change in |*V*_Th_| before and after mechanical deformation decreases, indicating improved mechanical stability of the semiconductor channel. Collectively, these results identify the 8:2 formulation as an optimal composition that achieves robust mechanical durability with minimal compromise in transistor performance.

Thus, these results demonstrate that the microstructure‐controlled 8:2 composition achieves an optimal balance between electrical properties and mechanical resilience, such as stretchability and cyclic stability. Additionally, ISNDs fabricated from this material demonstrate essential features of both long‐ and short‐term plasticity, including SNDP, SVDP, and SRDP, establishing multi‐timescale neuromorphic functions. This combination of robust mechanical properties and biologically relevant synaptic behavior establishes the 8:2 composition as a promising material platform for next‐generation on‐device neuromorphic computing under dynamic mechanical conditions.

## 4 Mechanical Durability and on‐device Reservoir Computing Performance of ISNDs Under Repeated Deformation

**FIGURE 4:**
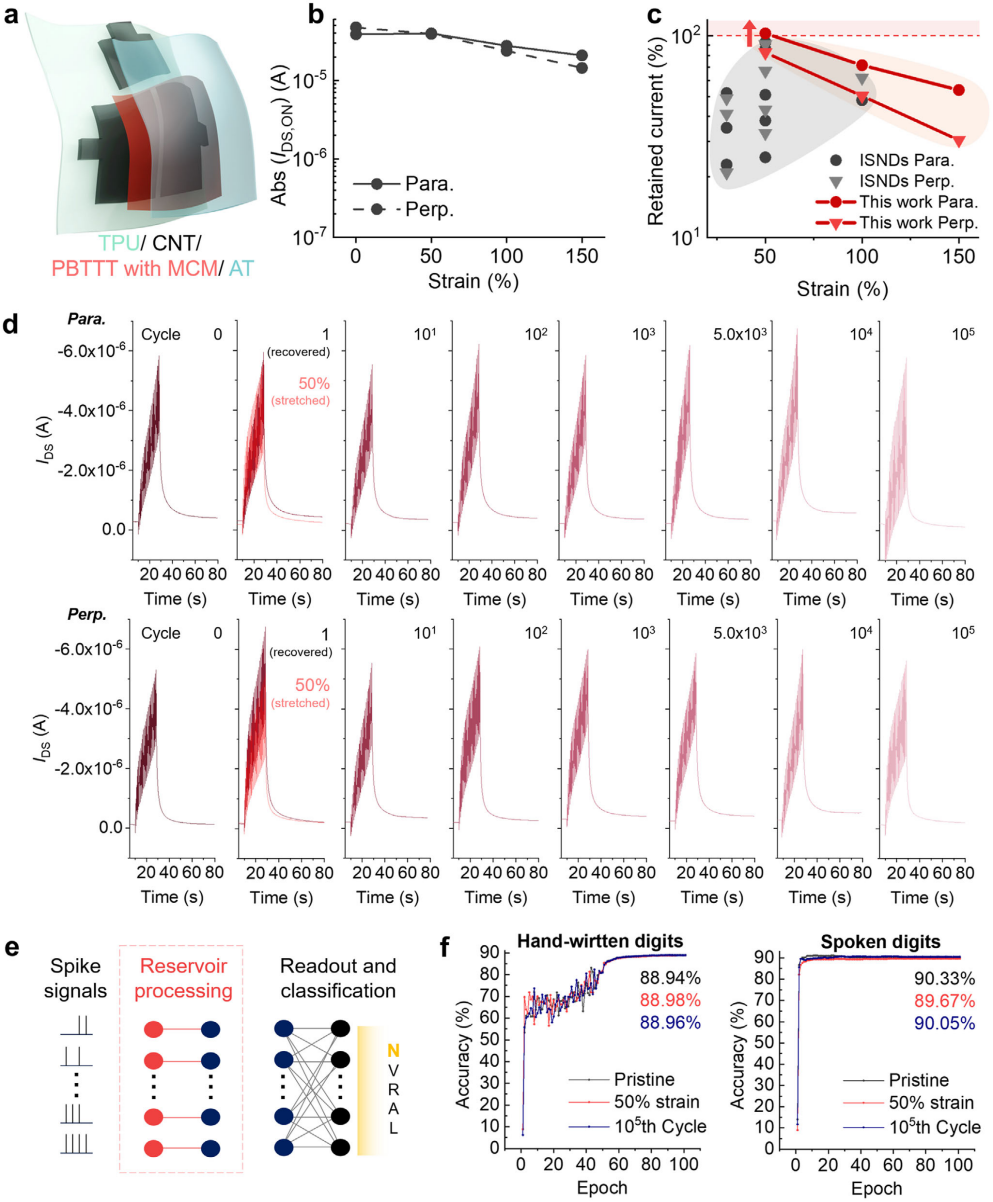
Mechanical durability and on‐device reservoir computing performance of ISNDs under repeated deformation. (a) Schematic of the ISND structure consisting of TPU substrate, CNT electrodes, PBTTT with MCM, and AT electrolyte. (b) *I*_DS_ measured under increasing strain up to 150%, applied parallel (Para.) and perpendicular (Perp.) to the channel length direction, showing minimal degradation in both directions. (c) Comparison plot of reported mechanical durability of ISNDs, highlighting the ultra‐high durability of our ISND using PBTTT with MCM. (d) Spike responses measured at 50% strain under repeated stretching cycles (from 0 to 10^5^). (e) Schematic of reservoir computing system architecture, where incoming spike signals are processed through physical ISND reservoirs and classified via a readout layer. (f) Classification accuracy of ISND‐based reservoir computing under three mechanical conditions (pristine, 50% strain, and after 10^5^ strain cycles). Accuracy remains stable at 88.96% for hand‐written digits and 90.05% for spoken digits, demonstrating high mechanical resilience and functional reliability.

To comprehensively evaluate the mechanical robustness and neuromorphic stability of ISNDs, we fabricated the devices incorporating PBTTT with MCM 8:2 semiconducting channels using electrolyte‐gating (Figure 4a). This device architecture leverages the molecularly engineered bundle‐to‐mesh microstructure transition in the active layer, enabling efficient stress dissipation under multidirectional stretching. This ISND operates by ion migration and electrochemical doping at the electrolyte/ organic semiconductor interface, where presynaptic voltage spikes modulate channel conductance in an analog and history‐dependent manner, directly coupling signal processing and synaptic retention within a single device. This ion‐mediated conductance modulation enables biomimetic synaptic behaviors with short‐term retention dominant synaptic plasticity, which is essential for sensory processing and pre‐processing in reservoir computing [30, 31].

Consistent with this operational mechanism, spike‐voltage‐dependent plasticity (SVDP) and spike‐rate‐dependent plasticity (SRDP) of ISND were quantitatively evaluated using paired‐pulse facilitation (PPF) measurements (Figure S35). The PPF index systematically varies with gate spike amplitude, demonstrating clear voltage‐dependent modulation of synaptic plasticity, while increasing inter‐spike interval leads to a gradual reduction in PPF, reflecting rate‐dependent synaptic relaxation governed by ionic diffusion dynamics.

The transfer characteristics under strain were first characterized by applying uniaxial tensile strain in two directions relative to the charge transport direction, parallel (Para.) and perpendicular (Perp.) (Figure 4b; Figure S36). In the parallel direction, the current increased by 2.8% at 50% strain, but decreased by 28.4% and 46.2% at 100% and 150%. In the perpendicular direction, it decreased by 17.3%, 49.5%, and 69.6% at the same strain levels. The ISNDs withstand up to 150% tensile strain in both orientations, and the minimal variation at 50% strain confirms stable charge transport pathways under mechanical deformation. Compared with previously reported ISNDs, our MCM‐incorporated devices exhibit superior current retention at 50% strain and sustain higher maximum stretchability, thereby surpassing prior counterparts in both mechanical and electrical properties. (Figure 4c; Table S3).

To evaluate mechanical durability under cyclic stretching, devices were subjected to up to 10^5^ cycles of tensile loading at 0% and 50% strain in both parallel and perpendicular orientations (Figure 4d). Synaptic responses were recorded under identical gate‐voltage stimuli (*V*_GS_ = −3.5 V, 300 ms duration, 300 ms interval, 30 pulses). The amplitude and decay profiles remained highly stable throughout the entire cycling process. The device was kept under ambient conditions for one week during the measurement. After 10^5^ cycles under 50% strain, the maximum variation in peak current was only ∼15% compared to the pristine state. These results indicate that the cohesive mesh‐like polymer network effectively prevents electrical degradation under extremely high cycling conditions. Moreover, even the stretched devices with 50% strain retained nearly identical current values to the unstretched state, with only ∼1% variation (Figure S37 and Table S4), confirming exceptional mechanical‐electrical stability.

Acting as nonlinear physical reservoirs, the devices transformed temporally coded spike inputs into distinct current states. Under all tested conditions (pristine, 50% strain, and after 10^5^ cycles under 50% strain), the devices exhibited clear multi‐level responses to all 16 possible 4‐bit spike sequences and consistent reservoir output currents with less than 16% variation, confirming robust temporal information encoding unaffected by mechanical stress (Figure S38). The classification performance of the RC system was quantified as two benchmark datasets: handwritten digits and spoken digits (Figure 4f; Figure S39). For handwritten digits classification (Figure 4f, left), the accuracy achieved after training was 88.94% (0% strain), 88.98% (50% strain), and 88.96% (10^5^ cycles), demonstrating negligible variation across all conditions. For spoken digit recognition (Figure 4f, right), accuracies of 90.33%, 89.67%, and 90.05% were achieved for the same respective conditions, again indicating remarkable mechanical invariance of the neuromorphic computing capability. The maintenance of classification accuracy even after extensive cycling under substantial strain highlights the intrinsic resilience of the MCM‐based channel material and the mechanical stability of the device platform. Thus, these findings demonstrate that the microstructure‐controlled 8:2 composition enables simultaneous achievement of mechanical stretchability, electrical durability, and neuromorphic computing functions. The ability to retain classification accuracy with a minimal variation and stable synaptic responses under both strain and repeated deformation underscores the potential of this material platform for robust, on‐device neuromorphic computing in mechanically dynamic environments.

To further assess the role of MCM in enabling mechanically robust neuromorphic operation, ISNDs using a conjugated polymer containing 0% MCM were fabricated and evaluated under tensile strain (Figure S40). Upon 50% stretching, the 0% MCM devices exhibited baseline current instability arising from crack opening in the active layer. Under these conditions, spike‐induced current modulation was no longer discernible, indicating the loss of continuous charge transport pathways and functional synaptic behavior. This mechanically induced failure highlights that, without MCM incorporation, the polymer film cannot accommodate tensile deformation while preserving electrically connected pathways required for spike‐based signal processing.

## 5 Conclusions

This study presents a breakthrough in achieving high device‐level mechanical durability in ISNDs through molecular engineering of SPs that preserves the rigid conjugated backbone moiety while incorporating a microstructure‐controlling moiety (MCM) to impart intrinsic stretchability and enhance mechanical robustness. Introducing an MCM into the polymer backbone modulates chain packing from a dense bundle‐like morphology to a more relaxed mesh‐like morphology, enabling the formation of long‐range and stable percolation networks that preserve charge transport pathways under strain. ISNDs fabricated with this material exhibit ultrahigh device‐level stretchability of 150% and exceptional cyclic durability of up to 10^5^ cycles at 50% strain, with less than 15% variation in output current while maintaining biologically plausible spike‐processing capabilities. This durability corresponds to approximately 1.36 years of continuous operation under repeated deformation at a rate of 200 cycles per day. Furthermore, reservoir computing simulations using these devices achieved consistent reservoir output currents with less than 16% variation, and well‐preserved classification accuracies with negligible variation for handwritten and spoken digit recognition, respectively, even under 50% strain and after 10^5^ mechanical cycles. This work offers a robust molecular design strategy that advances the practical utility of ISNDs, supporting their integration into on‐body computing platforms for healthcare monitoring and human‐like intelligent electronics.

## 6 Methods

### 6.1 Materials

2‐bromoethyl acrylate (stabilized with 900–1500 MCM of 4‐methoxyphenol) was purchased from Alfa Aesar. Bis(trifluoromethane)sulfonimide lithium salt (Li[TFSI]) and 2,2‐dimethoxy‐2‐phenylacetophenone (DMPA) were purchased from TCI chemicals. Dichloromethane (DCM) and acetonitrile were purchased from Daejung Industry. TPU (KA‐480) was obtained from Kolon Industries. CNT (P3‐SWNT) was purchased from Carbon Solutions. Poly(vinylidene fluoride‐co‐hexafluoropropylene) (PVDF‐HFP) (Mw = 400 000) was purchased from Sigma–Aldrich. Isopropanol alcohol (IPA) was purchased from Fisher Scientific. [EMIM][TFSI] was purchased from Solvionic. 1‐butylimidazole, poly(ethylene glycol) diacrylate (PEGDA, average Mn 250), SN, trichloroethylene, octadecyltrimethoxysilane (OTS), ammonium hydroxide, tetrahydrofuran (THF), and were purchased from Sigma–Aldrich. Polydimethylsiloxane (PDMS) was purchased from Dow Corning.

The 1‐[2‐acryloyloxyethyl]‐3‐butylimidazolium bis(trifluoromethane) sulfonimide (AT) monomer was synthesized following previously reported procedures. 2‐Bromoethyl acrylate (5.0 g, 1.0 equiv.) was mixed with 1‐butylimidazole (3.64 g, 1.05 equiv.) in 30 mL of acetonitrile. The reaction mixture was stirred at 60°C overnight. Upon completion, acetonitrile was removed under reduced pressure using a rotary evaporator. The resulting crude product, 1‐[2‐acryloyloxyethyl]‐3‐butylimidazolium bromide ([AEBI]Br), was dissolved in 30 mL of DI water. Subsequently, Li[TFSI] (8.0 g, 1.0 equiv.) was added to the aqueous solution, and the mixture was stirred at room temperature overnight to allow anion exchange. The resulting water‐insoluble AT monomer was extracted with DCM and washed with DI water at least three times. The organic layer was dried over anhydrous sodium sulfate, and the final product was obtained as a pale‐yellow liquid after vacuum drying (< 10^−^^1^ Torr).

A film was prepared with the following process. AT monomer was mixed with 2 mol% PEGDA as a crosslinker. Subsequently, 0.5 mol% DMPA dissolved in ethanol (100 mg mL^−1^) was added as the photoinitiator. The resulting solution was stirred thoroughly, and ethanol was removed under vacuum (< 10^−^^1^ Torr) at room temperature. The viscous mixture was then cast into a polytetrafluoroethylene mold, separated by polyethylene terephthalate spacers. Photopolymerization was carried out by irradiating 365 nm UV light for 30 min. After curing, unreacted monomers were removed by immersing the cured AT in DCM overnight. The final elastomer was dried in a vacuum oven at 60°C for 24 h to eliminate any residual solvent.

### 6.2 Rigid Electrolyte‐gated Transistor With Spin Coating and Contact Transfer Methods

*OTS treatment of Si/SiO_2_ wafer*: A solution of OTS (1 µg mL^−^^1^) was spin‐coated onto UV ozone‐treated Si/SiO_2_ wafers at 2000 rpm for 30 s. The coated substrates were then placed in a vacuum desiccator for 12 h along with a vial containing ammonium hydroxide solution. Finally, the substrates were rinsed with toluene to remove any residual OTS molecules.

*Rigid electrolyte‐gated transistor with spin coating*: The channel electrodes were fabricated by thermal evaporation of Au 30 nm/ Cr 5 nm on glass. The semiconducting polymer solutions were spin‐coated, then annealed at 150°C for 60 min. The PVDF‐HFP solid‐state electrolyte film was transferred onto the semiconductor films by a cut‐and‐paste method.

*Rigid electrolyte‐gated transistor with contact transfer*: The channel electrodes were fabricated by thermal evaporation of Au 30 nm/ Cr 5 nm on glass. The semiconducting polymer solutions were spin‐coated on OTS‐treated Si/SiO_2_ wafer at 1000 rpm for 60 s. This semiconductor film was then transferred to channel electrodes by the PDMS contact transfer method. The PVDF‐HFP solid‐state electrolyte film was transferred onto the semiconductor films by a cut‐and‐paste method.

### 6.3 ISND Fabrication

80 mg of CNT powder was dispersed in 300 mL of mixed solvent of DI water and IPA at a volume ratio of 1:9. Next, the CNT solution was spray‐coated on a 120°C‐heated Si/SiO_2_ wafer. Then, TPU solutions were poured, dried overnight, and the films were peeled off from the substrate. The SP films fabricated on OTS‐treated Si/SiO_2_ wafer were transferred to CNT S/D electrodes by PDMS contact transfer method. Next, the AT polyelectrolyte film was transferred onto the semiconductor films by a cut‐and‐paste method.

### 6.4 Characterization of Films and Devices

The microstructure images were measured using atomic force microscopy (AFM) (NX‐10, Park System). The absorbance was measured using ultraviolet (UV) absorption spectroscopy (Lambda 465, PerkinElmer). The electrical properties of the devices were measured using a semiconductor analyzer (Keithley B1500A, Keysight). Grazing‐incident wide‐angle X‐ray scattering (GIWAXS) measurements were performed using a Xeuss 2.0 system (Xenocs, National Instrumentation Center for Environmental Management, Seoul National University) equipped with a Cu Kα X‐ray source (λ = 1.54189 Å). The sample‐to‐detector distance was fixed at 110 mm, and the scattering patterns were collected using a Dectris PILATUS 300 K detector.

### 6.5 Reservoir Computing Simulation

All simulations were conducted in a Python 3.7 environment. The handwritten digit recognition task was tested using the MNIST dataset (784 pixels per image). Each image was converted into 196 pulse trains, with each train encoding four pixels. The spoken digit recognition task was tested using FSDD. The raw audio file from each dataset was converted to 64 × 80 pixels by using the Lyons hearing model. The converted data were binarized after normalization and grouped into four pixels of 1280 (spoken digit) pulse trains. These pulse trains were fed as input to the reservoir nodes and used to train and test a single‐layer readout (MNIST: 196 input and 10 output neurons; spoken digit: 1280 input and 10 output neurons) that was simulated using the CrossSim Ver 2.0 (Sandia National Laboratory, USA).

## Conflicts of Interest

The authors declare no conflicts of interest.

## Supporting information




**Supporting File**: smll73524‐sup‐0001‐SuppMat.pdf.

## Data Availability

The data that support the findings of this study are available from the corresponding author upon reasonable request.
