# Supplementary material for: Mechanically Durable Intrinsically Stretchable Neuromorphic Devices via Molecular Microstructure Design
Source: Small. 2026 Jul 10;22(41):e12071. doi: 10.1002/smll.202512071 (PMC13392780; doi:10.1002/smll.202512071)
Supplement: Supplementary file 1 — Supporting File: smll73524‐sup‐0001‐SuppMat.pdf. [file SMLL-22-e12071-s001.pdf]

Supporting information for

Mechanically durable intrinsically stretchable neuromorphic devices via molecular microstructure design

Kwan-Nyeong Kim[1†], Ho-Eon Baek[2†], Min-Jun Choi[3†], Min-Jun Sung[1], Seung-Woo Lee[1], Chae-Yun Song[1], Hyun-Haeng Lee[1], Sangjun Ma[1] Karina Ayu Larasati[2], Jeong-Yun Sun[1,4], Hea-Lim Park[3], Yun-Hi Kim[2]*,and Tae-Woo Lee[1,4,5,6]**

[1]Department of Materials Science and Engineering, Seoul National University, Seoul 08826, Republic of Korea.

[2]Department of Chemistry and RIMA, Gyeongsang National University, Jinju 52828, Republic of Korea.

[3]Department of Materials Science and Engineering, Seoul National University of Science and Technology, Seoul 01811, Republic of Korea.

[4]Research Institute of Advanced Materials, Seoul National University, Seoul, 08826, Republic of Korea.

[5]Department of Materials Science and Engineering, Interdisciplinary Program in Bioengineering, Institute of Engineering Research, Soft Foundry, Seoul National University, Seoul National University, Seoul, 08826, Republic of Korea.

[6]SN Display Co., Ltd. Seoul, 08826, Republic of Korea.

Corresponding author. Email : twlees@snu.ac.kr, ykim@gnu.ac.kr, parkhl21@seoultech.ac.kr

The PDF file includes:

Notes S1

Fig. S1 to S40

Tables S1 to S4

Table of Contents

Note S1. Material synthesis

Fig. S1. ^{1}H NMR data of 3-dodecylthiophene (1)

Fig. S2. ^{13}C NMR data of 3-dodecylthiophene (1).

Fig. S3. Q-TOF+ MASS data of 3-dodecylthiophene (1)

Fig. S4. ^{1}H NMR data of 4,4'-didodecyl-2,2'-bithiophene (2)

Fig. S5. ^{13}C NMR data of 4,4'-didodecyl-2,2'-bithiophene (2)

Fig. S6. Q-TOF+ MASS data of 4,4'-didodecyl-2,2'-bithiophene (2)

Fig. S7. ^{1}H NMR data of 5,5'-dibromo-4,4'-didodecyl-2,2'-bithiophene (3)

Fig. S8. ^{13}C NMR data of 5,5'-dibromo-4,4'-didodecyl-2,2'-bithiophene (3)

Fig. S9. Q-TOF+ MASS data of 5,5'-dibromo-4,4'-didodecyl-2,2'-bithiophene (3)

Fig. S10. ^{1}H NMR data of 1,8-di(thiophen-2-yl)octane (4)

Fig. S11. ^{1}H NMR data of gDPP-TTVTT Polymer

Fig. S12. Q-TOF+ MASS data of 1,8-di(thiophen-2-yl)octane (4)

Fig. S13. ^{1}H NMR data of 1,8-bis(5-(trimethylstannyl)thiophen-2-yl)octane (5)

Fig. S14. ^{13}C NMR data of 1,8-bis(5-(trimethylstannyl)thiophen-2-yl)octane (5)

Fig. S15. Q-TOF+ MASS data of 1,8-bis(5-(trimethylstannyl)thiophen-2-yl)octane (5)

Fig. S16. ^{1}H NMR data PBTTT-T8T (6a). X:Y=10:1

Fig. S17. Gel permeation chromatogram of the PBTTT-T8T (10:1) polymer run in chloroform solution

Fig. S18. ^{1}H NMR data PBTTT-T8T (6b). X:Y = 9:1

Fig. S19. Gel permeation chromatogram of the PBTTT-T8T (9:1) polymer run in chloroform solution

Fig. S20. ^{1}H NMR data PBTTT-T8T (6c). X:Y = 8:2

Fig. S21. Gel permeation chromatogram of the PBTTT-T8T (8:2) polymer run in chloroform solution

Fig. S22. ^{1}H NMR data PBTTT-T8T (6d). X:Y=7:3

Fig. S23. Gel permeation chromatogram of the PBTTT-T8T (7:3) polymer run in chloroform solution

Fig. S24. 1D AFM topography of 8:2 and 7:3

Fig. S25. AFM phase images

Fig. S26. Raman spectroscopy analysis

Fig. S27. Grazing incident wide angle X-ray scattering (GIWAXS) analysis

Fig. S28. Cyclic voltammetry of PBTTT with MCM moiety

Fig. S29. Thermogravimetric analysis (TGA) of PBTTT with MCM moiety

Fig. S30. Threshold voltage and hysteresis in transfer curves according to MCM ratio

Fig. S31. Spike responses according to MCM ratio

Fig. S32. Decay current measurements after spike response

Fig. S33. SVDP and SRDP of MCM ratio 8:2 polymer

Fig. S34. Transconductance (gm) values and relative changes after cyclic stretching.

Fig. S35. SVDP and SRDP of ISND using MCM ratio 8:2 polymer

Fig. S36. Transfer curves of ISND with MCM 8:2 polymer under strain

Fig. S37. Comparison plot of reported stretchability in spike response of ISNDs

Fig. S38. Spike responses and multistate of ISND physical reservoir

Fig. S39. Color confusion matrix plots

Fig. S40. Spike response of ISND using 10:0

Table S1. The structural information obtained from GIWAXS 1D profiles

Table S2. Electrochemical properties of PBTTT with MCM moiety

Table S3. Comparison of mechanical and electrical performance in transfer curves of ISNDs incorporating organic semiconductors and solid gel electrolytes

Table S4. Comparison of mechanical and electrical performance in spike responses of ISNDs incorporating organic semiconductors and solid gel electrolytes

Note S1. Materials synthesis

Synthesis of 3-dodecylthiophene (1)

In a nitrogen-substituted 1000 mL three-neck round-bottom flask, magnesium turnings (7.45 g, 306.68 mmol) and anhydrous tetrahydrofuran (400 mL) were added, and 1-bromooctane (64.15 g, 245.35 mmol) was added dropwise. After the addition was complete, the mixture was refluxed for 3 h and then transferred to a solution of 3-bromothiophene (20.00 g, 122.67 mmol) and Pd(dppf)Cl_2 (0.36 g, 0.49 mmol) at 0°C. The resulting solution was refluxed overnight under a nitrogen atmosphere. After cooling down to room temperature and 500 mL of water was added. The product was extracted with ethyl acetate, followed by dark yellow solution. The ethyl acetate layer was dried over $MgSO_4$ and evaporated under reduced pressure. The residue was then purified by column chromatography on silica gel using hexane to obtain a colorless liquid. (22.10 g, 71%) ^{1}H NMR (300 MHz, $CDCl_3$) δ (MCM) = 7.26 (dd, J = 4.9, 3.0 Hz, 1H), 7.00 – 6.92 (m, 2H), 2.70 – 2.59 (m, 2H), 1.64 (dq, J = 11.7, 6.9 Hz, 2H), 1.37 – 1.27 (m, 18H), 0.96 – 0.86 (m, 3H). ^{13}C NMR (300 MHz, $CDCl_3$) δ 143.3, 128.3, 125.0, 119.8, 77.5, 77.2, 77.0, 76.6, 31.9, 30.6, 30.3, 29.7, 29.7, 29.6, 29.5, 29.4, 22.7, 14.1. HRMS (QTof, m/z): calcd for $C_{16}H_{28}S$, 252.1912; found 253.1981.

Synthesis of 4,4'-didodecyl-2,2'-bithiophene (2)

In a nitrogen-substituted 500 mL three-neck round-bottom flask, 3-dodecylthiophene (22.00 g, 87.14 mmol) and anhydrous tetrahydrofuran (180 mL) were added and the temperature was lowered to -78°C, followed by stirring for 15 minutes. To this solution, n-BuLi (2.5 M in hexanes, 38.83 mL, 97.08 mmol) was added dropwise. The mixture was then gradually warmed to room temperature and stirred for 1 h. After cooling to -78°C, $CuCl_2$ (14.53 g, 108.06 mmol) was added

in a single portion. The reaction mixture was stirred overnight, allowing the temperature to gradually rise to room temperature. The product was extracted with ethyl acetate, followed by yellow solution. The ethyl acetate layer was dried over $MgSO_4$ and evaporated under reduced pressure. The residue was then purified by column chromatography on silica gel using hexane to obtain a light-yellow gel. (11.87 g, 54%) ^{1}H NMR (300 MHz, $CDCl_3$) δ (MCM) = 7.27-7.25 (dd, J = 4.90, 2.95 Hz, 1H), 6.97-6.94 (m, 2H), 2.67-2.62 (m, 2H), 1.67-1.55 (m, 2H), 1.32-1.30 (m, 24H). ^{13}C NMR (300 MHz, CDCl3) δ 144.0, 143.3, 137.4, 128.3, 125.0, 124.8, 119.7, 118.7, 77.5, 77.2, 77.0, 76.6, 31.9, 30.6, 30.5, 30.4, 30.3, 29.7, 29.7, 29.6, 29.5, 29.4, 29.3, 22.7, 14.2. HRMS (QTof, m/z): calcd for $C_{32}H_{54}S_2$, 502.3667; found 502.3664.

Synthesis of 5,5'-dibromo-4,4'-didodecyl-2,2'-bithiophene (3)

In a nitrogen-substituted 1000 mL three-neck round-bottom flask, 4,4'-didodecyl-2,2'-bithiophene (11.00 g, 21.87 mmol), acetic acid (150 mL), and chloroform (150 mL) were added and stirred. In a dark environment, n-bromosuccinimide (7.86 g, 44.18 mmol) was added, and the mixture was stirred overnight at room temperature. The product was extracted with ethyl acetate, followed by yellow solution. The ethyl acetate layer was dried over $MgSO_4$ and evaporated under reduced pressure. The residue was then purified by column chromatography on silica gel using hexane to obtain light yellow solid. (8.90 g, 62%) ^{1}H NMR (300 MHz, $CDCl_3$) δ (MCM) = 6.79 (s, 2H), 2.56-2.51 (m, 4H), 1.64-1.58 (m, 4H), 1.35-1.29 (m, 39H), 0.93-0.88 (m, 6H). ^{13}C NMR (300 MHz, CDCl3) δ 143.0, 136.1, 124.5, 107.9, 31.9, 29.7, 29.7, 29.6, 29.6, 29.5, 29.4, 29.4, 29.2, 22.7, 14.2. HRMS (QTof, m/z): calcd for $C_{32}H_{52}Br_2S_2$, 658.1877; found 660.1882.

Synthesis of 1,8-di(thiophen-2-yl)octane (4)

Dissolve thiophene (10.00 g, 118.85 mmol) in 80 mL of anhydrous tetrahydrofuran in an oven-dried flask. Cool the solution to -40°C using a dry-ice-isopropanol bath, then add n-BuLi (2.5 M in hexane, 35.65 mL, 89.13 mmol) dropwise. Maintain the solution in the cooling bath for 30 minutes, then allow it to warm to room temperature and stir for an additional 30 minutes. Re-cool the solution to -40°C. Add dibromooctane (8.08 g, 29.71 mmol) dropwise using a syringe. Gradually warm the mixture to room temperature by removing the cooling bath and stir the mixture overnight. The product was extracted with ethyl acetate, followed by yellow solution. The ethyl acetate layer was dried over $MgSO_4$ and evaporated under reduced pressure. The residue was then purified by column chromatography on silica gel using hexane to obtain colorless liquid. (6.52 g, 79%) ^{1}H NMR (300 MHz, $CDCl_3$) δ (MCM) = 7.14-7.12 (m, 2H), 6.95-6.93 (m, 2H), 6.81-6.79 (m,2H), 2.87-2.82 (t, J = 7.62 Hz, 4H), 1.73-1.68 (m, 4H), 1.47-1.36 (m, 10H). ^{13}C NMR (300 MHz, $CDCl_3$) δ 145.8, 126.6, 123.9, 123.9, 122.8, 122.7, 77.5, 77.0, 76.6,

34.0, 32.8, 31.8, 31.7, 29.9, 29.3, 29.2, 29.1, 29.0, 28.7, 28.1. HRMS (QTof, m/z): calcd for $C_{16}H_{22}S_2$, 278.1163; found 279.1250.

Synthesis of 1,8-bis(5-(trimethylstannyl)thiophen-2-yl)octane (5)

In a nitrogen-substituted 500 mL three-neck round-bottom flask, dissolve 1,8-di(thiophen-2-yl)octane (6.00 g, 21.55 mmol) in 150 mL of anhydrous tetrahydrofuran. Cool the mixture to -40°C, then add n-BuLi (2.5 M in hexane, 21.55 mL, 53.87 mmol) dropwise and stir at the same temperature for 1 h. Subsequently, cool the mixture to -70°C, then add trimethyltin chloride (12.88 g, 64.64 mmol) in 50 mL of THF dropwise. Gradually warm the mixture to room temperature and stir overnight. The product was extracted with ether, followed by dark yellow solution. The ether layer was dried over $MgSO_4$ and evaporated under reduced pressure. The residue was briefly filtered through alumina silica and then recrystallized from petroleum ether by white solid. (4.02 g, 31%) ^1H NMR (300 MHz, $CDCl_3$) δ (MCM) = 6.90-6.89 (d, J = 3.17 Hz, 2H), 6.78-6.77 (d, J = 3.16 Hz, 2H), 2.76-2.70 (t, J = 7.60 Hz, 4H), 1.58-1.53 (m, 4H), 1.27-1.14 (m, 8H). ^{13}C NMR (300 MHz, CD_2Cl_2) δ 151.9, 151.8, 151.7, 135.2, 135.0, 134.8, 134.6, 125.7, 125.4, 125.1, 54.2, 54.0, 53.8, 53.6, 53.4, 53.3, 53.1, 52.7, 32.0, 29.9, 29.6, 29.3, 29.2, -6.2, -6.3, -8.7, -11.0, -11.1. HRMS (QTof, m/z): calcd for $C_{22}H_{38}S_2Sn_2$, 606.0459; found 606.0392.

Synthesis of PBTTT-T8T (6a-d)

6a: In a nitrogen-purged 20 mL round-bottom flask, 5,5'-dibromo-4,4'-didodecyl-2,2'-bithiophene (200.00 mg 0.30 mmol), and 2,5-bis(trimethylstannyl)thieno[3,2-b]thiophene (141.01 mg, 0.30 mmol) were dissolved in 3 mL of anhydrous chlorobenzene and bubbled for 30 minutes. Then, tris(dibenzylideneacetone)dipalladium(0) (8.32 mg, 0.01 mmol) and Tri(o-tolyl)phosphine (11.06 mg, 0.04 mmol) were added, and the mixture was refluxed and stirred at 120°C for 24h. After the reaction was complete, 2-Bromothiophene (mg, mmol) dissolved in 0.5 mL of chlorobenzene was added and refluxed for 30min to end-capping the reaction. The mixture was then cooled to room temperature, and the product was precipitated by adding 200 mL of methanol and filtered. The polymer was subsequently subjected to Soxhlet extraction and washed sequentially with methanol, acetone, hexane, toluene, and chloroform. The resulting solution was concentrated by evaporation and dissolved in chloroform. The product was then precipitated by adding 200 mL of methanol and filtered to obtain a dark red solid. (0.10 g, 52%) ^1H NMR (300 MHz, $CDCl_3$) δ (MCM) = 7.08-7.03 (m, 4H), 2.81-2.78 (m, 4H), 2.56-2.55 (m, 2H), 1.72-1.29 (m, 6H), 1.28-1.29 (m, 68H), 0.92-0.88 (m, 12H). GPC (Chloroform) M_n = 8 kDa, M_w = 11 kDa, PDI = 1.29

6b: In a nitrogen-purged 20 mL round-bottom flask, 5,5'-dibromo-4,4'-didodecyl-2,2'-

bithiophene (200.00 mg 0.30 mmol), 2,5-bis(trimethylstannyl)thieno[3,2-b]thiophene (126.91 mg, 0.27 mmol), and 1,8-bis(5-(trimethylstannyl)thiophen-2-yl)octane (18.29 mg, 0.03 mmol) were dissolved in 3 mL of anhydrous chlorobenzene and bubbled for 30 minutes. Then, tris(dibenzylideneacetone)dipalladium(0) (8.32 mg, 0.01 mmol) and Tri(o-tolyl)phosphine (11.06 mg, 0.04 mmol) were added, and the mixture was refluxed and stirred at 120°C for 24h. After the reaction was complete, 2-Bromothiophene (mg, mmol) dissolved in 0.5 mL of chlorobenzene was added and refluxed for 30min to end-capping the reaction. The mixture was then cooled to room temperature, and the product was precipitated by adding 200 mL of methanol and filtered. The polymer was subsequently subjected to Soxhlet extraction and washed sequentially with methanol, acetone, hexane, toluene, and chloroform. The resulting solution was concentrated by evaporation and dissolved in chloroform. The product was then precipitated by adding 200 mL of methanol and filtered to obtain a dark red solid. (0.10 g, 51%) ^{1}H NMR (300 MHz, $CDCl_3$) δ (MCM) = 7.06-6.72 (m, 4H), 2.81-2.78 (m, 4H), 2.55-2.37 (m, 1H), 1.70-1.69 (m, 4H), 1.29-1.28 (m, 50H), 0.92-0.88 (m, 8H) GPC (Chloroform) M_n = 10 kDa, M_w = 14 kDa, PDI = 1.39

6c: In a nitrogen-purged 20 mL round-bottom flask, 5,5'-dibromo-4,4'-didodecyl-2,2'-bithiophene (200.00 mg 0.30 mmol), 2,5-bis(trimethylstannyl)thieno[3,2-b]thiophene (112.7 mg, 0.242 mmol), and 1,8-bis(5-(trimethylstannyl)thiophen-2-yl)octane (36.89 mg, 0.06 mmol) were dissolved in 3 mL of anhydrous chlorobenzene and bubbled for 30 minutes. Then, tris(dibenzylideneacetone)dipalladium(0) (8.32 mg, 0.01 mmol) and Tri(o-tolyl)phosphine (11.06 mg, 0.04 mmol) were added, and the mixture was refluxed and stirred at 120°C for 24h. After the reaction was complete, 2-Bromothiophene (mg, mmol) dissolved in 0.5 mL of chlorobenzene was added and refluxed for 30min to end-capping the reaction. The mixture was then cooled to room temperature, and the product was precipitated by adding 200 mL of methanol and filtered. The polymer was subsequently subjected to Soxhlet extraction and washed sequentially with methanol, acetone, hexane, toluene, and chloroform. The resulting solution was concentrated by evaporation and dissolved in chloroform. The product was then precipitated by adding 200 mL of methanol and filtered to obtain a dark red solid. (0.10 g, 49%) ^{1}H NMR (300 MHz, $CDCl_3$) δ (MCM) = 7.09-6.74 (m, 4H), 2.83-2.78 (m, 5H), 2.56-2.54 (m, 1H), 1.70-1.69 (m, 5H), 1.39-1.37 (m, 50H), 0.92-0.88 (m, 8H). GPC (Chloroform) M_n = 70 kDa, M_w = 307 kDa, PDI = 4.3

6d: In a nitrogen-purged 20 mL round-bottom flask, 5,5'-dibromo-4,4'-didodecyl-2,2'-bithiophene (200.00 mg 0.30 mmol), 2,5-bis(trimethylstannyl)thieno[3,2-b]thiophene (98.71 mg, 0.21 mmol), and 1,8-bis(5-(trimethylstannyl)thiophen-2-yl)octane (54.86 mg, 0.09 mmol) were dissolved in 3 mL of anhydrous chlorobenzene and bubbled for 30 minutes. Then, tris(dibenzylideneacetone)dipalladium(0) (8.32 mg, 0.01 mmol) and Tri(o-tolyl)phosphine

(11.06 mg, 0.04 mmol) were added, and the mixture was refluxed and stirred at 120°C for 24h. After the reaction was complete, 2-Bromothiophene (mg, mmol) dissolved in 0.5 mL of chlorobenzene was added and refluxed for 30min to end-capping the reaction. The mixture was then cooled to room temperature, and the product was precipitated by adding 200 mL of methanol and filtered. The polymer was subsequently subjected to Soxhlet extraction and washed sequentially with methanol, acetone, hexane, toluene, and chloroform. The resulting solution was concentrated by evaporation and dissolved in chloroform. The product was then precipitated by adding 200 mL of methanol and filtered to obtain a dark red solid. (0.10 g, 49%) ^1H NMR (300 MHz, $CDCl_3$) δ (MCM) = 7.06-6.74 (m, 4H), 2.83-2.78 (m, 5H), 2.56-2.54 (m, 1H), 1.70-1.69 (m, 5H), 1.39-1.37 (m, 50H), 0.92-0.88 (m, 8H). GPC (Chloroform) M_n = 15 kDa, M_w = 23 kDa, PDI = 1.50

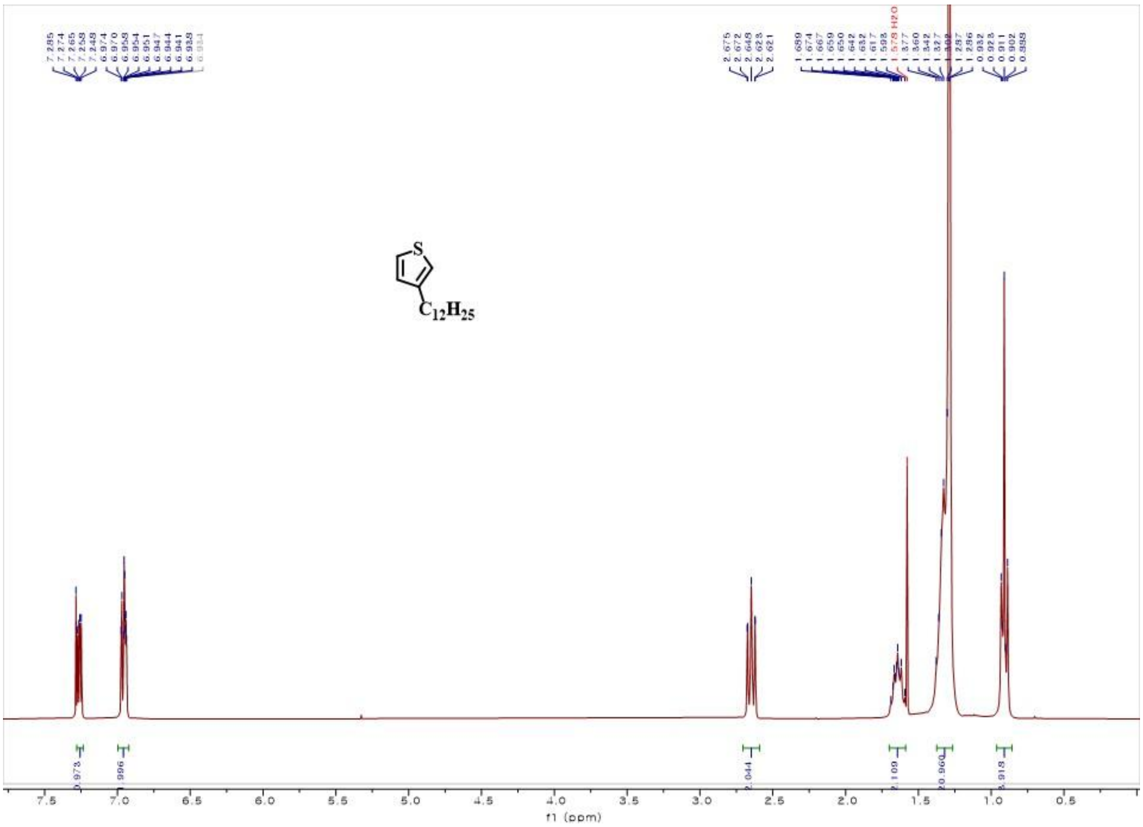

Figure S1. ^{1}H NMR data of 3-dodecylthiophene (1)

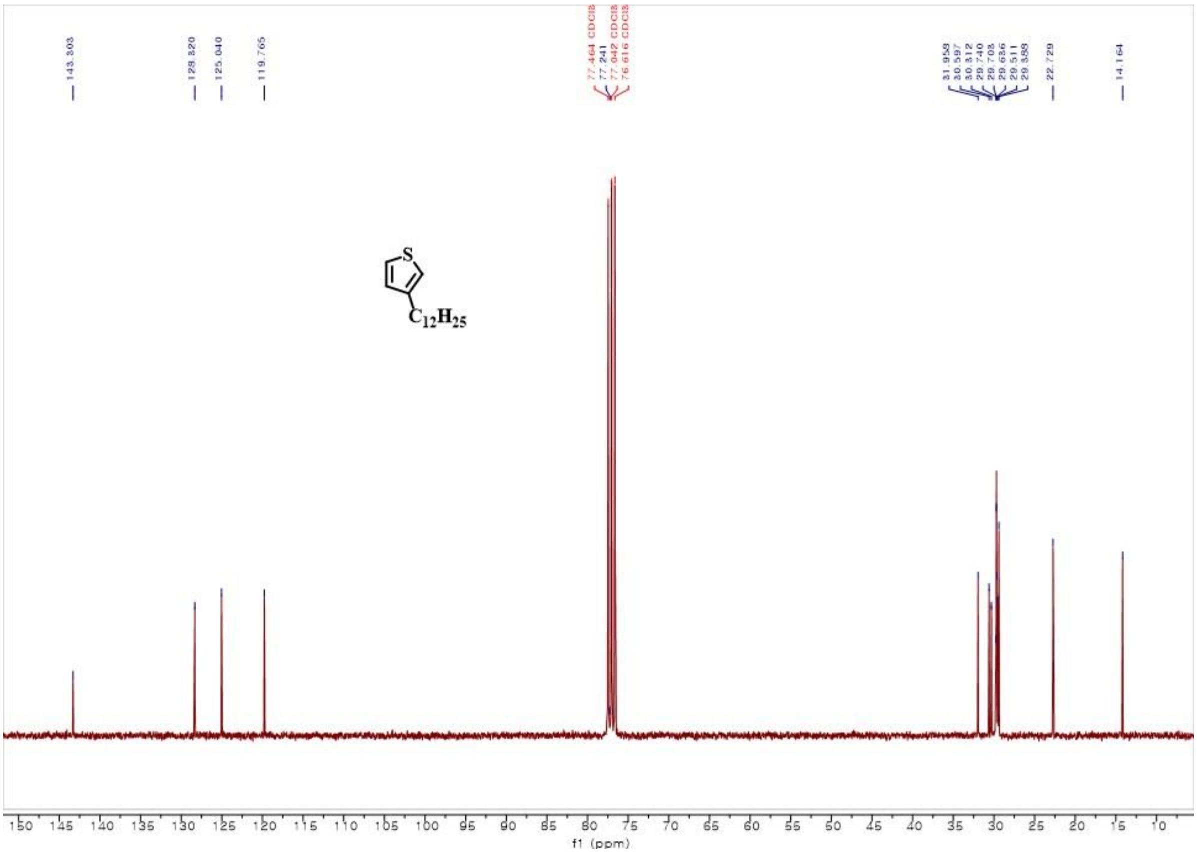

Figure S2. ^{13}C NMR data of 3-dodecylthiophene (1)

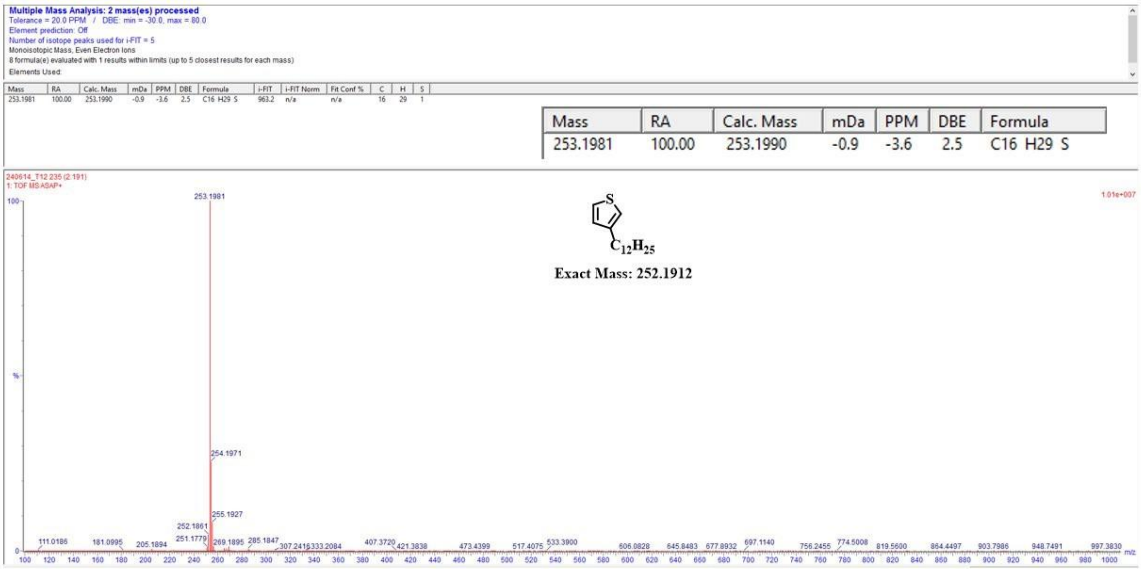

Figure S3. Q-TOF+ MASS data of 3-dodecylthiophene (1)

.

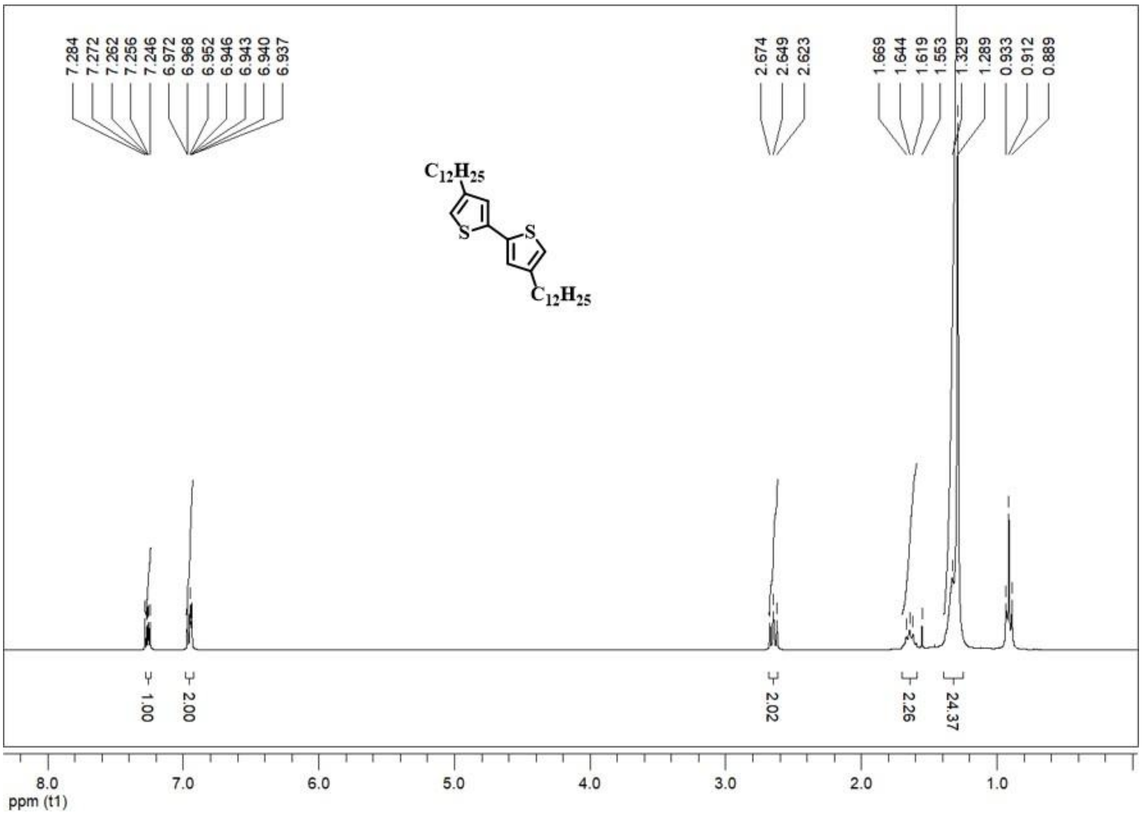

Figure S4. ^{1}H NMR data of 4,4'-didodecyl-2,2'-bithiophene (2)

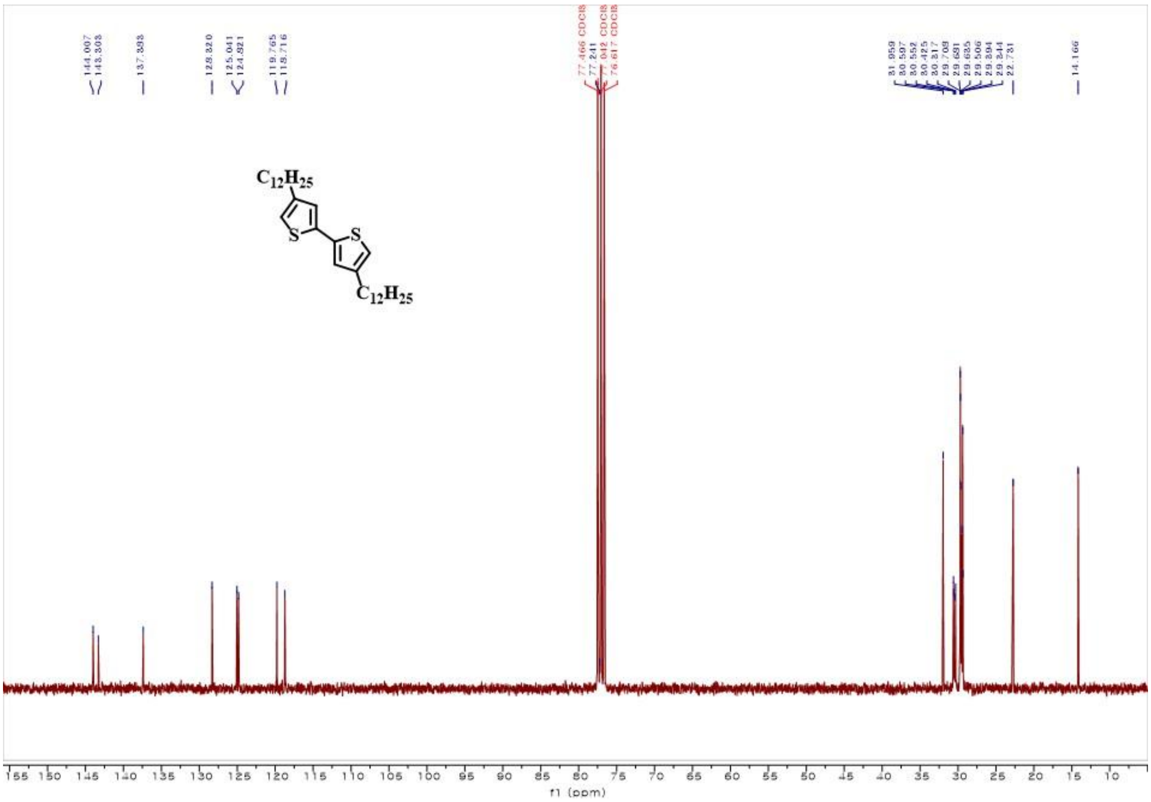

Figure S5. ^{13}C NMR data of 4,4'-didodecyl-2,2'-bithiophene (2)

.

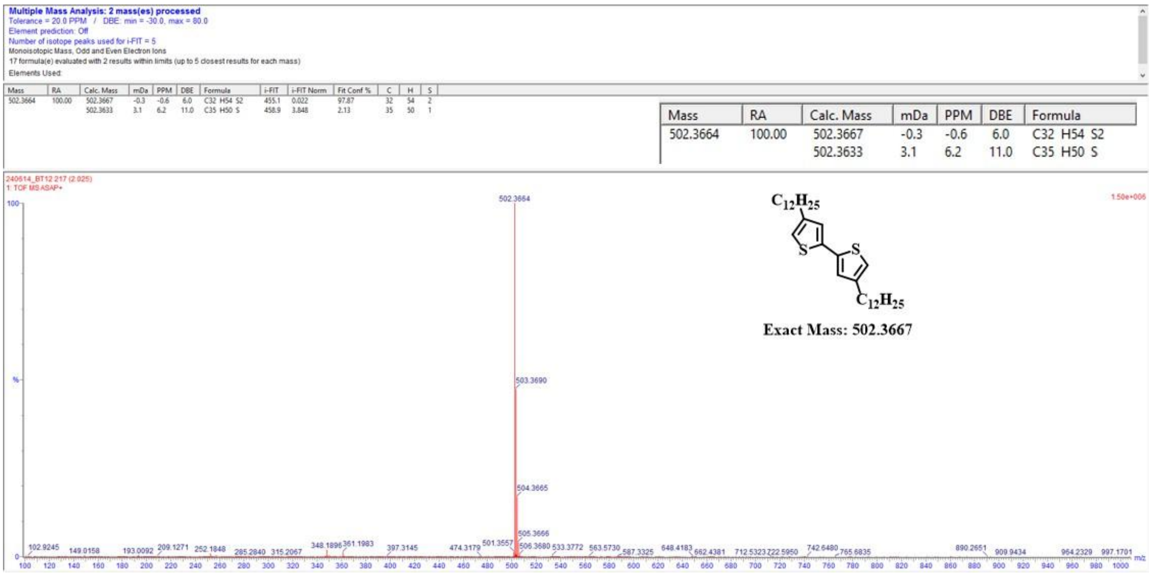

Figure S6. Q-TOF+ MASS data of 4,4'-didodecyl-2,2'-bithiophene (2)

.

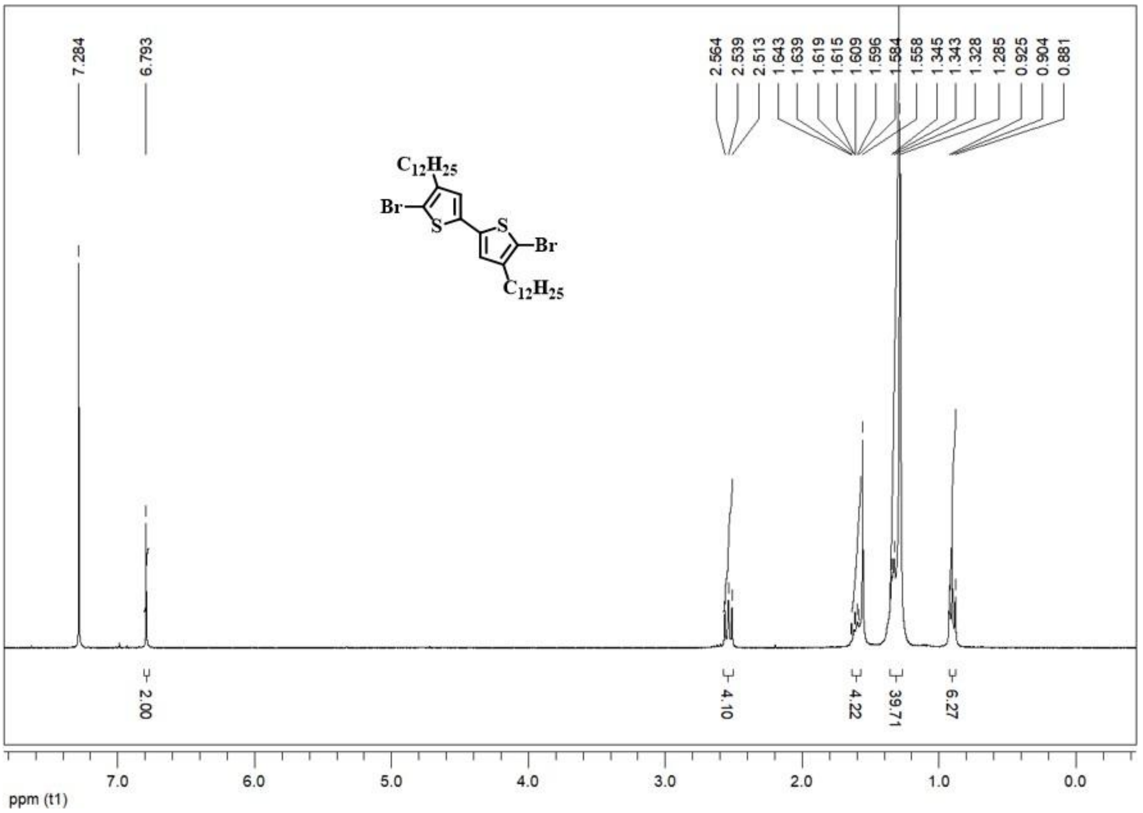

Figure S7. ^{1}H NMR data of 5,5'-dibromo-4,4'-didodecyl-2,2'-bithiophene (3)

.

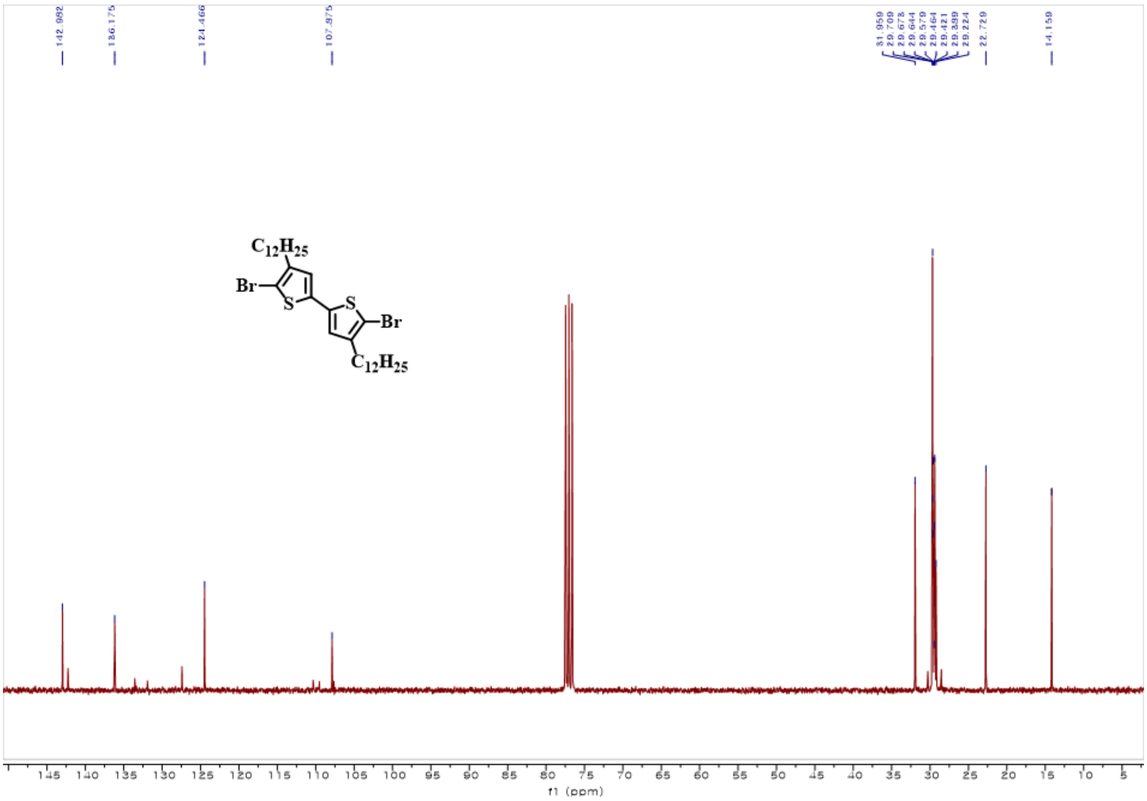

Figure S8. ^{13}C NMR data of 5,5'-dibromo-4,4'-didodecyl-2,2'-bithiophene (3).

.

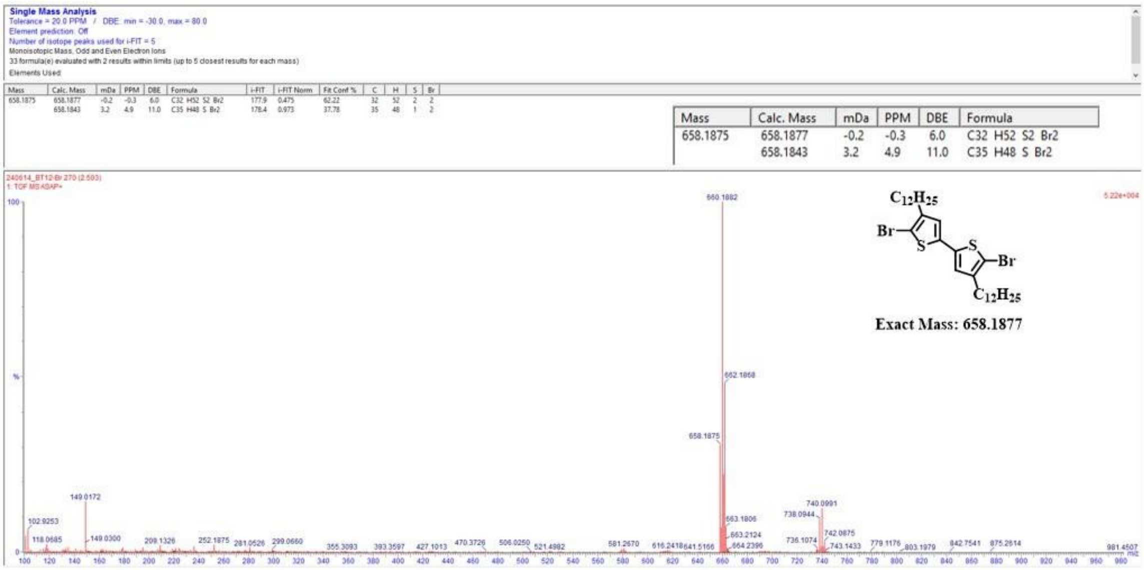

Figure S9. Q-TOF+ MASS data of 5,5'-dibromo-4,4'-didodecyl-2,2'-bithiophene (3)

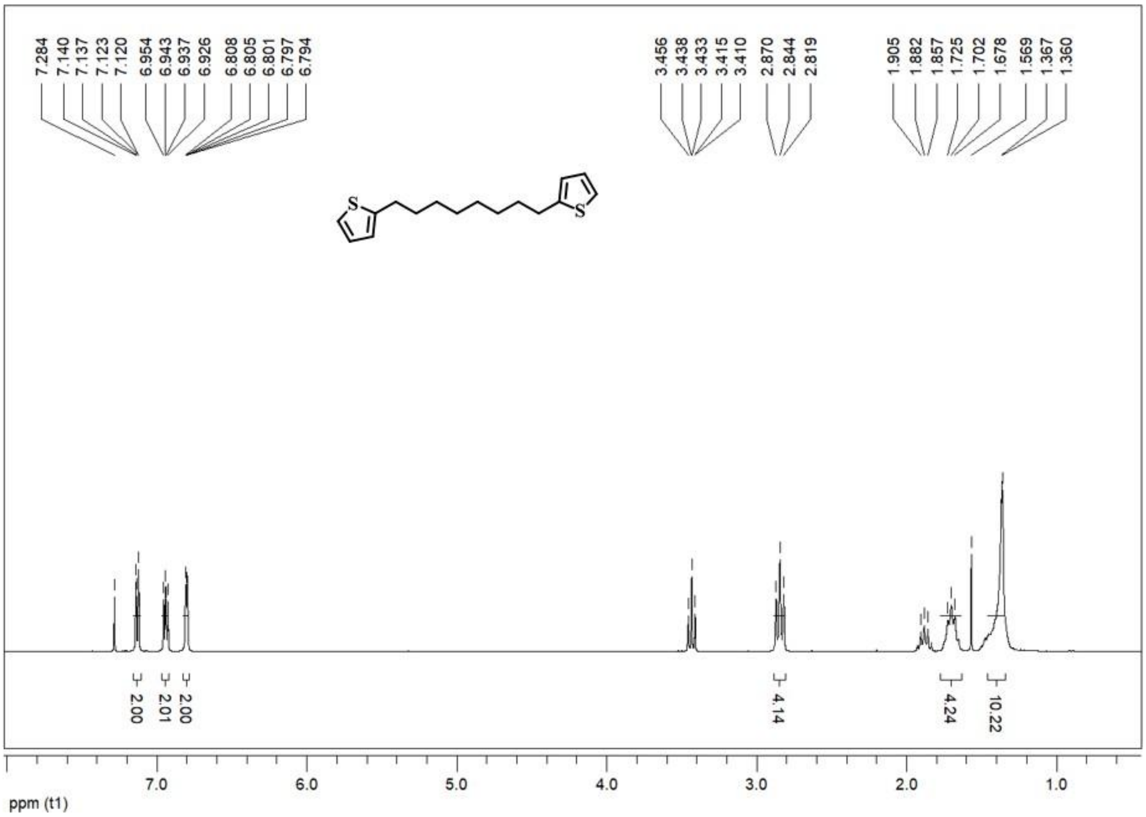

Figure S10. ^{1}H NMR data of 1,8-di(thiophen-2-yl)octane (4)

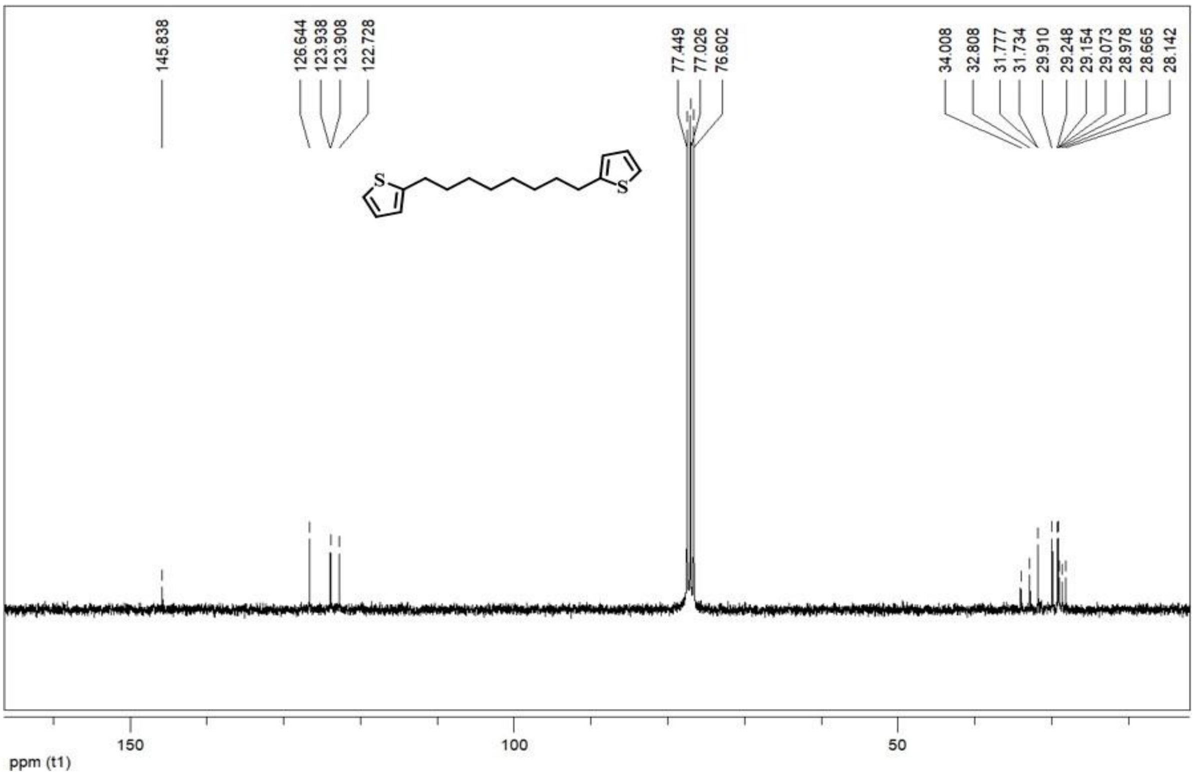

Figure S11. ^{1}H NMR data of gDPP-TTVTT Polymer.

.

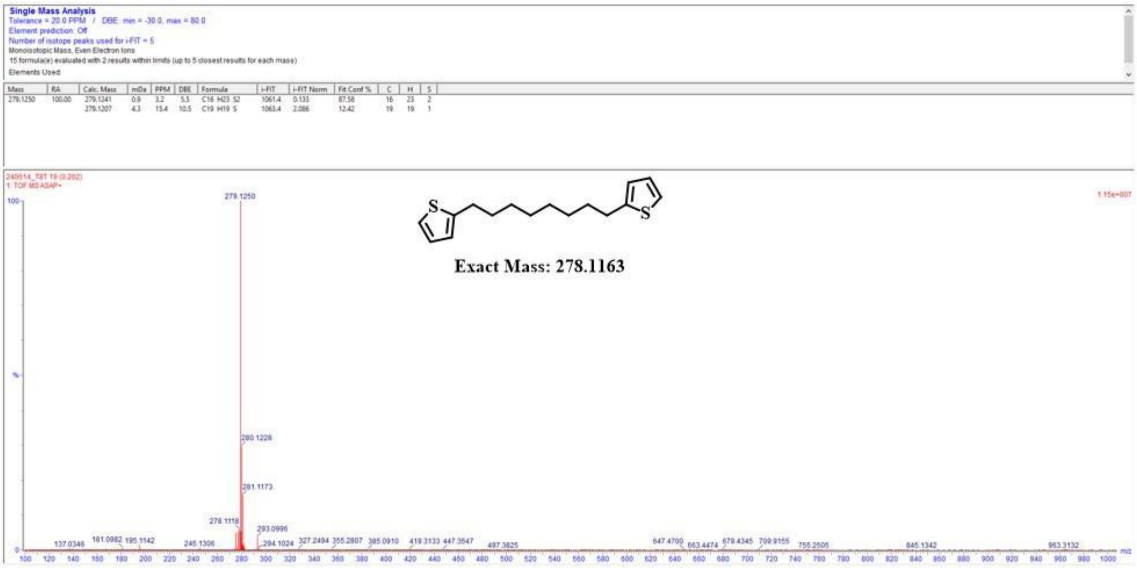

Figure S12. Q-TOF+ MASS data of 1,8-di(thiophen-2-yl)octane (4)

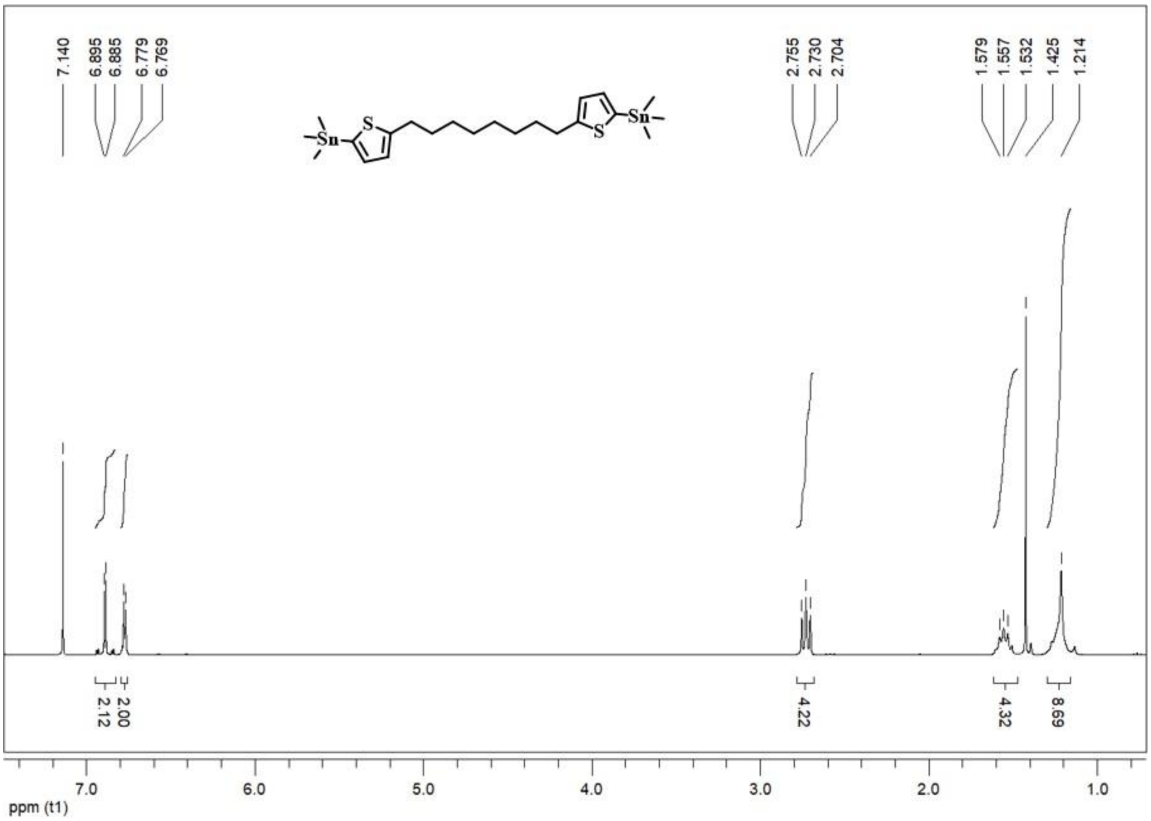

Figure S13. ^{1}H NMR data of 1,8-bis(5-(trimethylstannyl)thiophen-2-yl)octane (5)

.

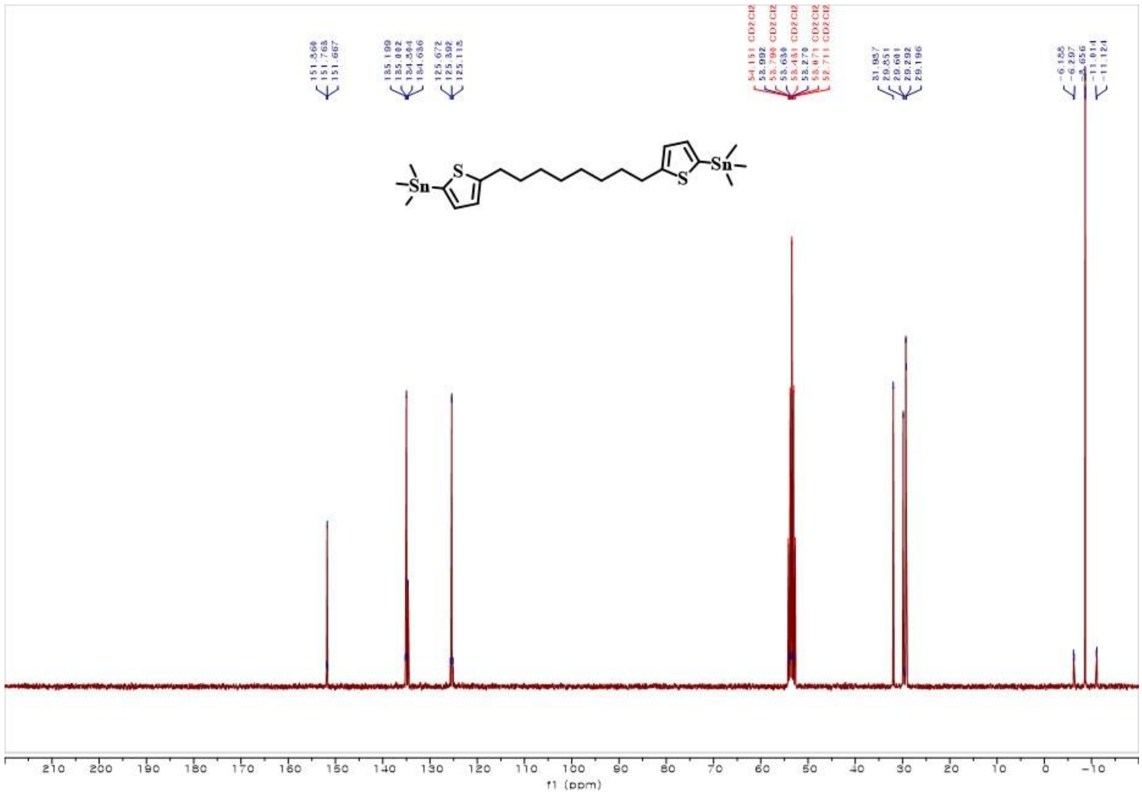

Figure S14. ^{13}C NMR data of 1,8-bis(5-(trimethylstannyl)thiophen-2-yl)octane (5).

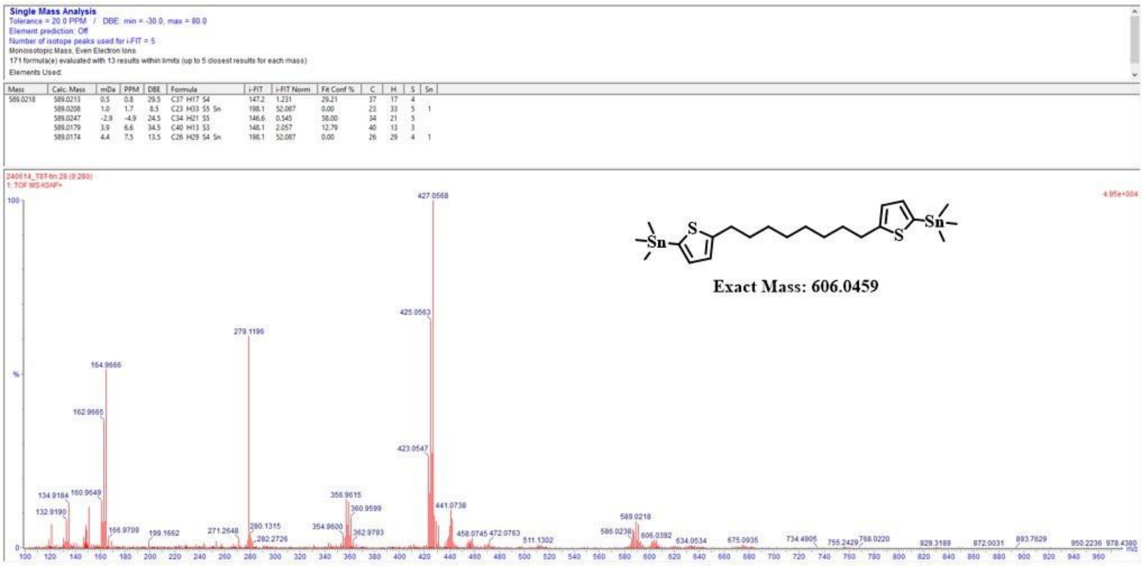

Figure S15. Q-TOF+ MASS data of 1,8-bis(5-(trimethylstannyl)thiophen-2-yl)octane (5)

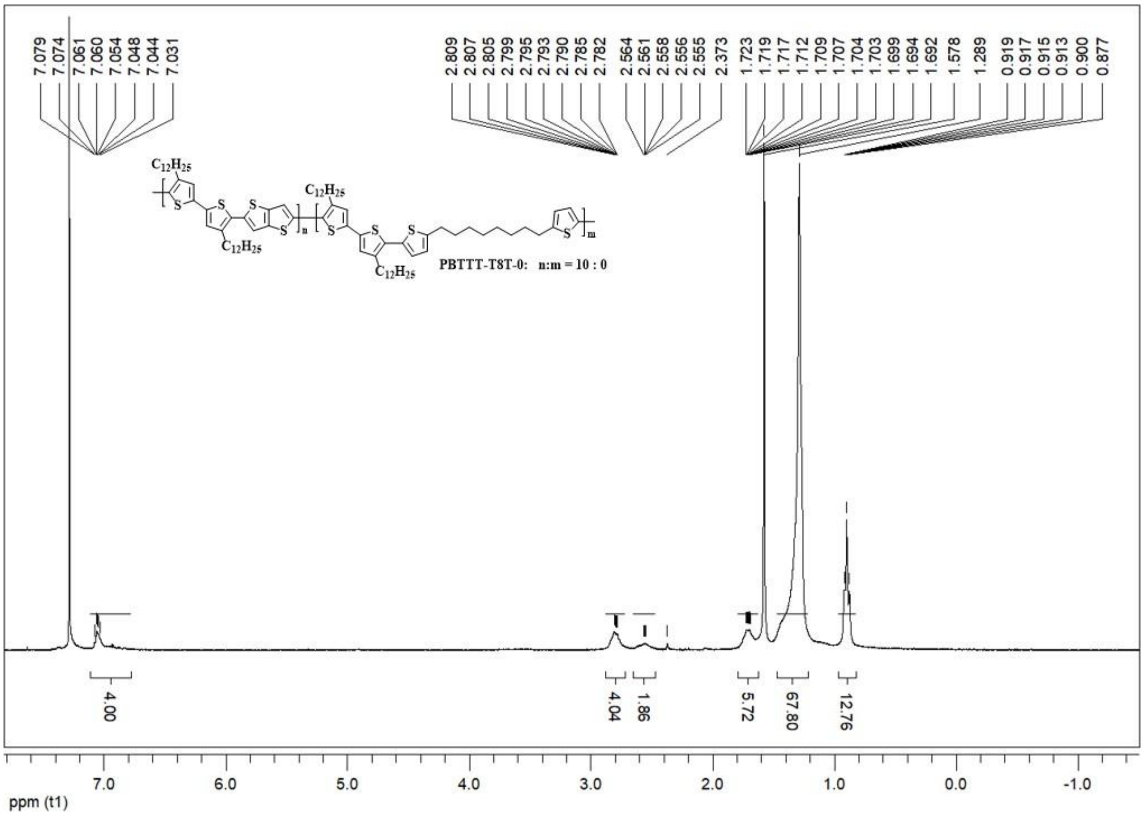

Figure S16. ^{1}H NMR data PBTTT-T8T (6a). X:Y=10:1

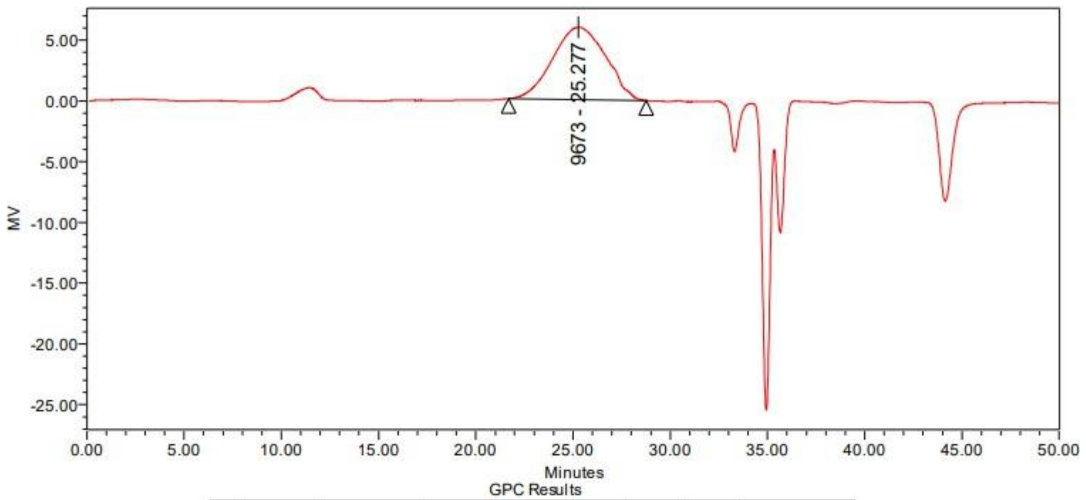

GPC Results

	Dist Name	Retention Time (min)	% Area	% Height	Mn	Mw	MP	Polydispersity
1		25.277	100.00	100.00	8068	10464	9673	1.296917

Figure S17. Gel permeation chromatogram of the PBTTT-T8T (10:1) polymer run in chloroform solution.

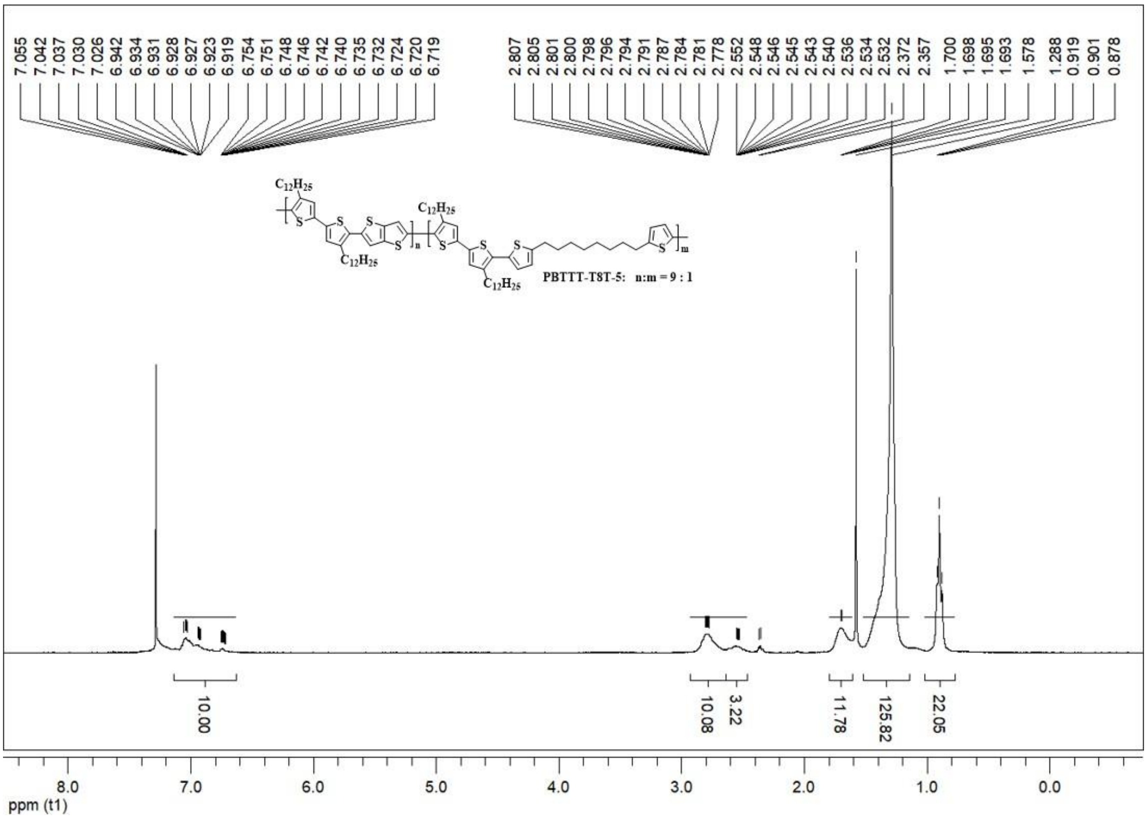

Figure S18. ^{1}H NMR data PBTTT-T8T (6b). X:Y = 9:1

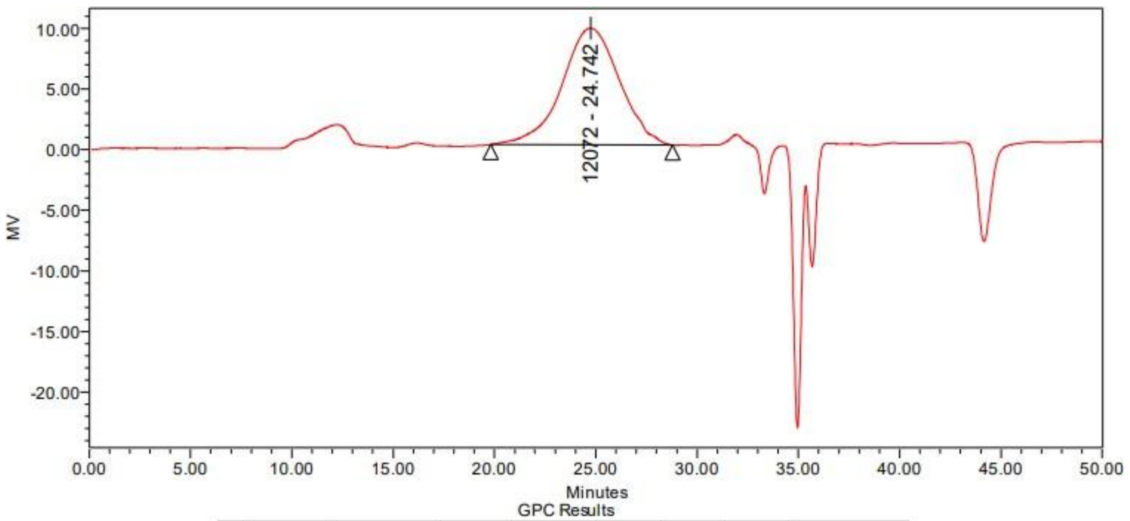

GPC Results

	Dist Name	Retention Time (min)	% Area	% Height	Mn	Mw	MP	Polydispersity
1		24.742	100.00	100.00	10041	13949	12072	1.389289

Figure S19. Gel permeation chromatogram of the PBTTT-T8T (9:1) polymer run in chloroform solution

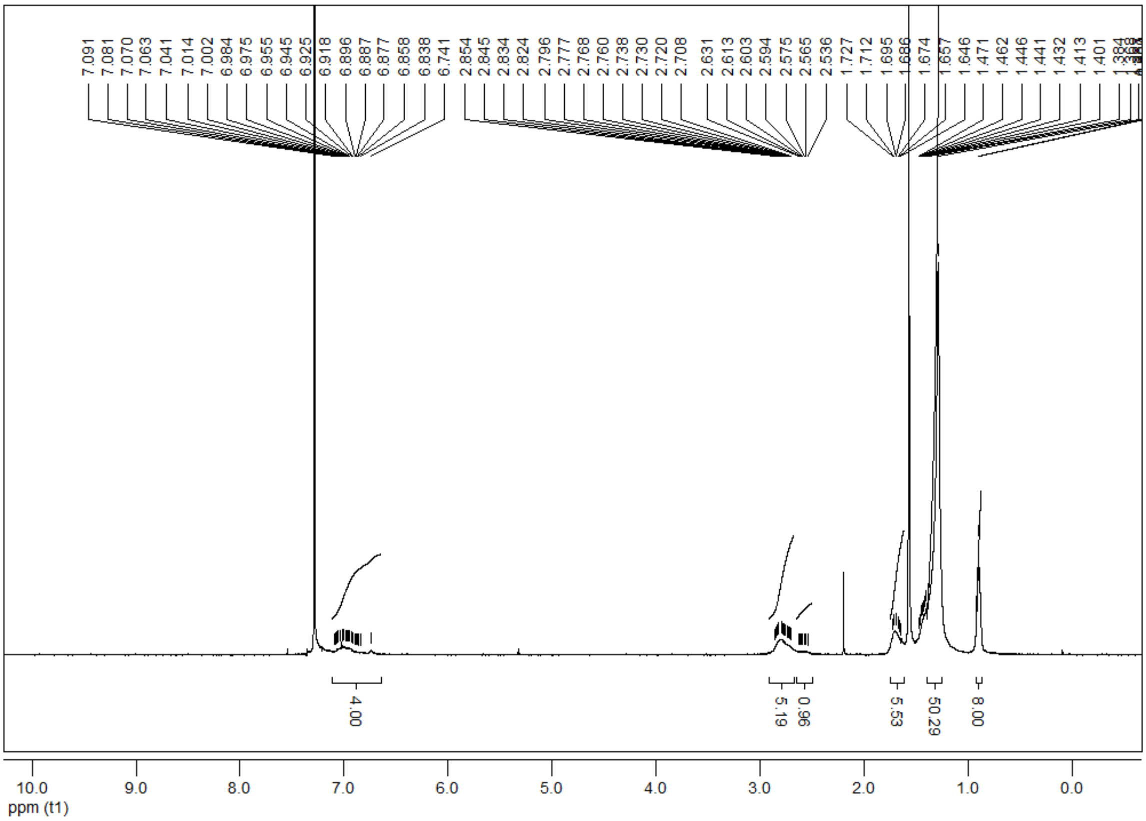

Figure S20. ^{1}H NMR data PBTTT-T8T (6c). X:Y = 8:2

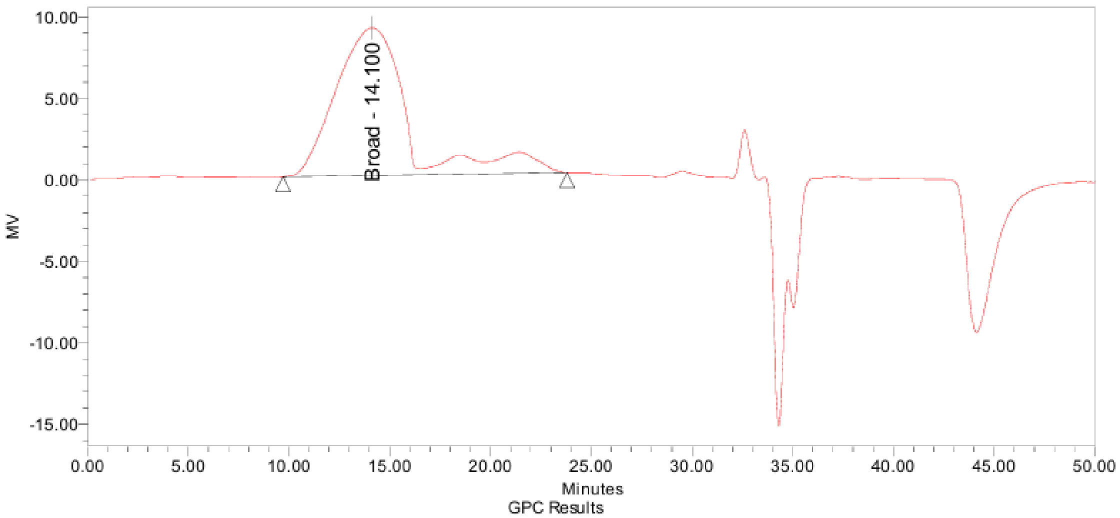

GPC Results

	Dist Name	Retention Time (min)	% Area	% Height	Mn	Mw	MP	Polydispersity
1		14.100	100.00	100.00	70020	307604		4.393082

Figure S21. Gel permeation chromatogram of the PBTTT-T8T (8:2) polymer run in chloroform solution

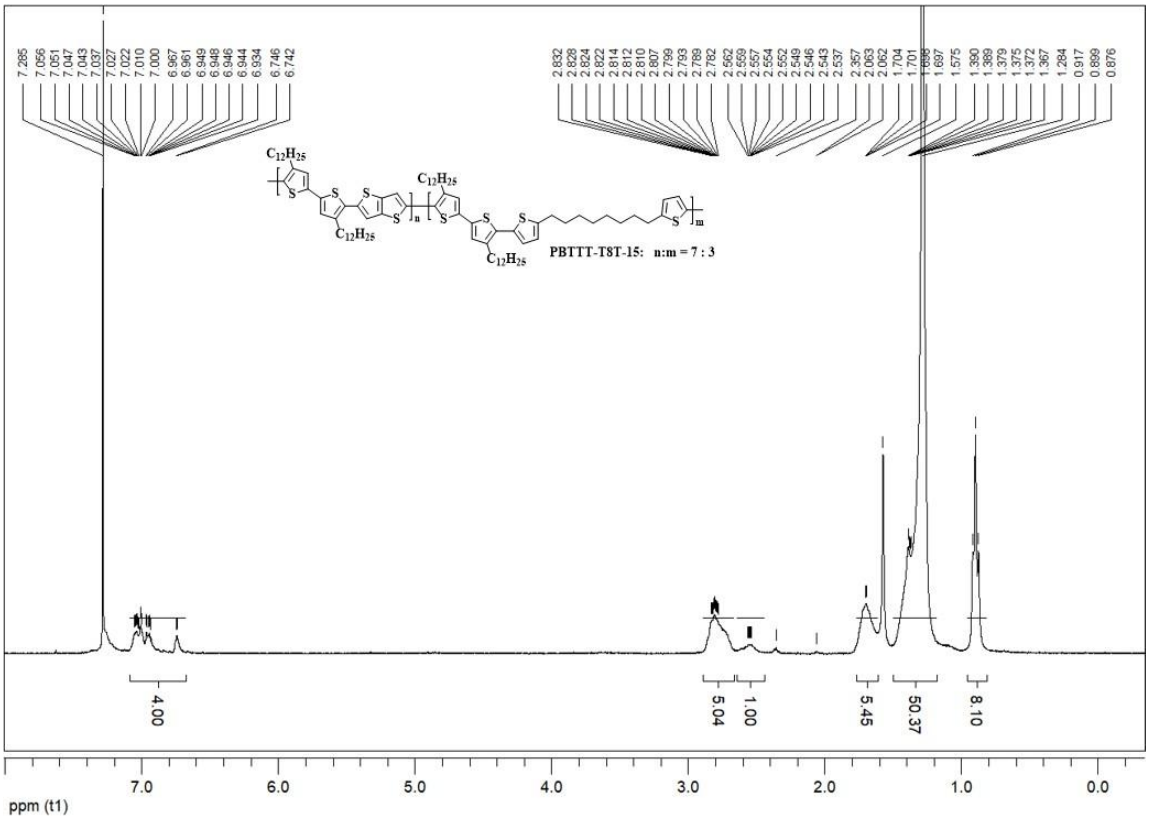

Figure. S22. ^{1}H NMR data PBTTT-T8T (6d). X:Y=7:3

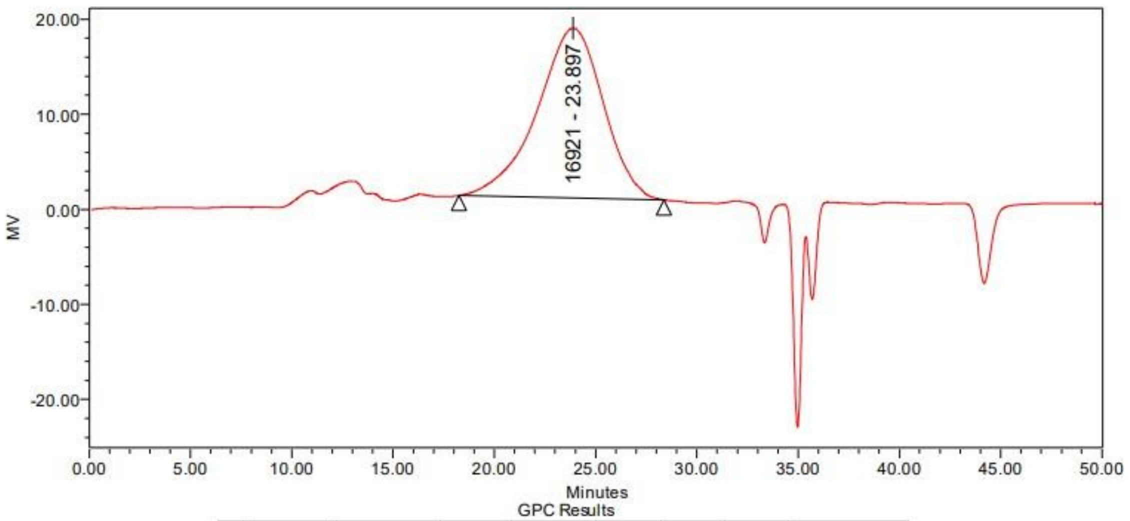

GPC Results

	Dist Name	Retention Time (min)	% Area	% Height	Mn	Mw	MP	Polydispersity
1		23.897	100.00	100.00	15164	22845	16921	1.506508

Figure S23. Gel permeation chromatogram of the PBTTT-T8T (7:3) polymer run in chloroform solution.

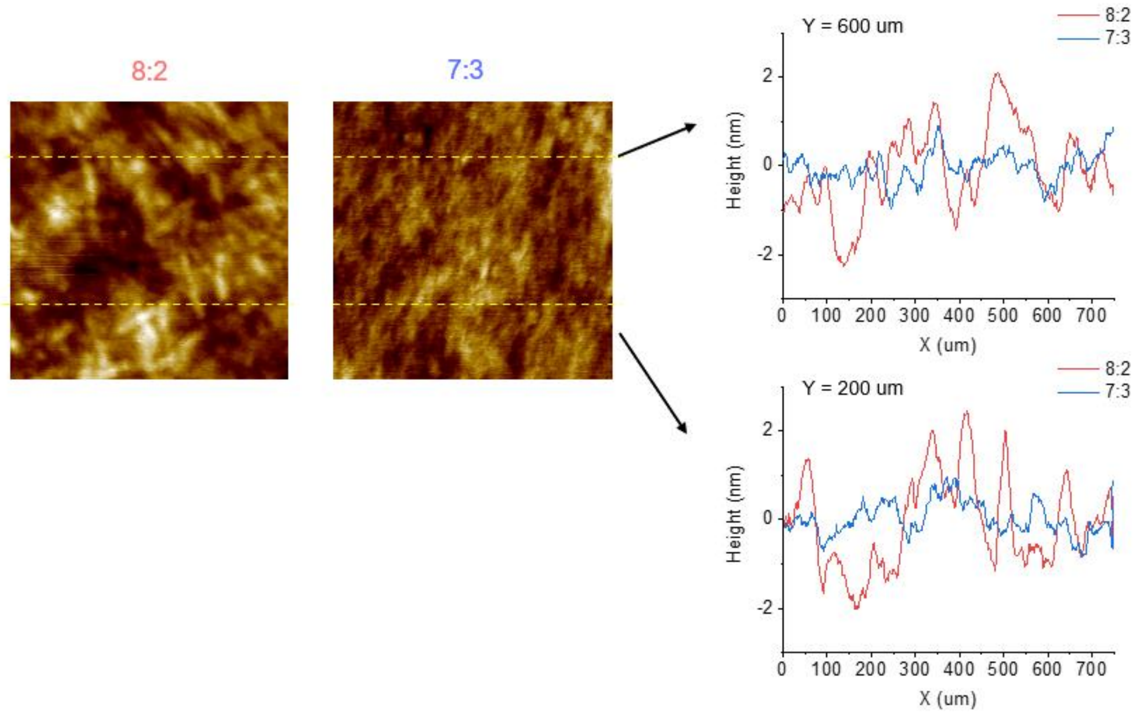

Figure S24. 1D AFM topography of 8:2 and 7:3.

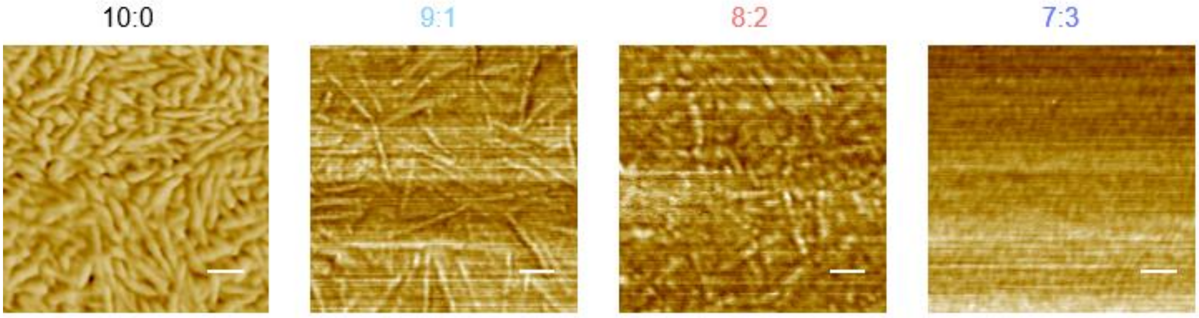

Figure S25. AFM phase images.

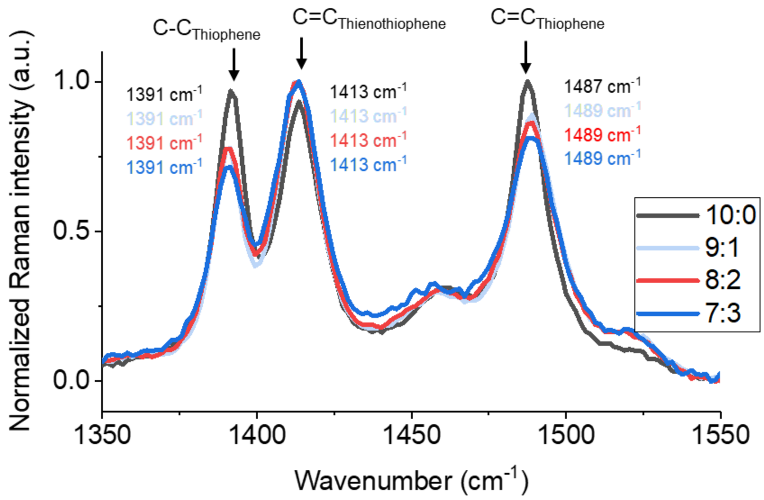

Figure S26. Raman spectroscopy analysis.

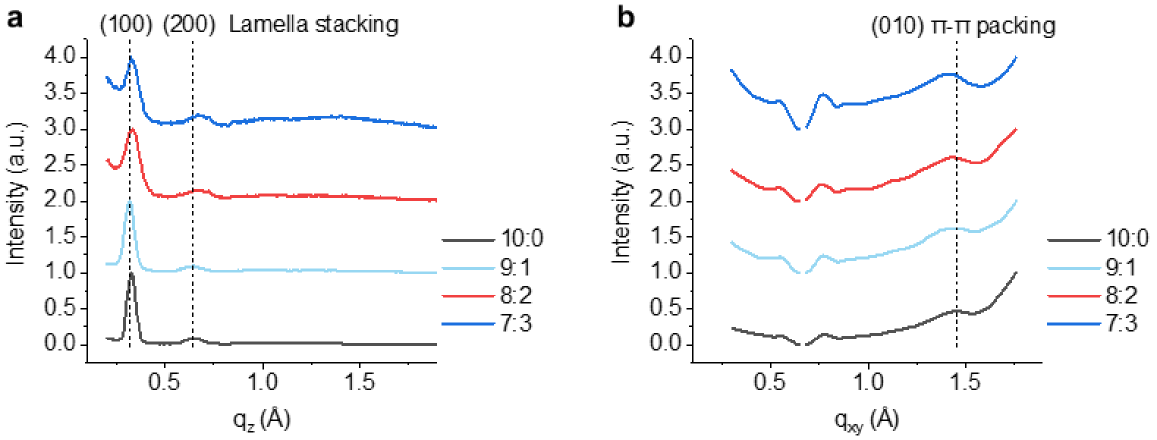

Figure S27. Grazing incident wide angle X-ray scattering (GIWAXS) analysis

MCM ratio	$\Delta Q_{(h00)}$ (Å^{-1})	$Q_{(010)}$ (Å^{-1})	$d_{(h00)}$ (Å)	$d_{(010)}$ (Å)	*FWHM Q (200)* (Å^{-1})	L_c (nm)
7:3	0.317	1.405	19.825	4.473	0.392	3.609
8:2	0.329	1.429	19.091	4.396	0.320	4.425
9:1	0.329	1.441	19.091	4.359	0.266	5.315
10:0	0.341	1.459	18.409	4.306	0.053	26.620

Table S1. The structural information obtained from GIWAXS 1D profiles. Coherence length (L_c) = κλ/βcosθ, κ = 0.9, λ = 1.54189 Å (the wavelength of the X-ray), β: FWHM width of the diffraction peak, θ: diffraction angle.

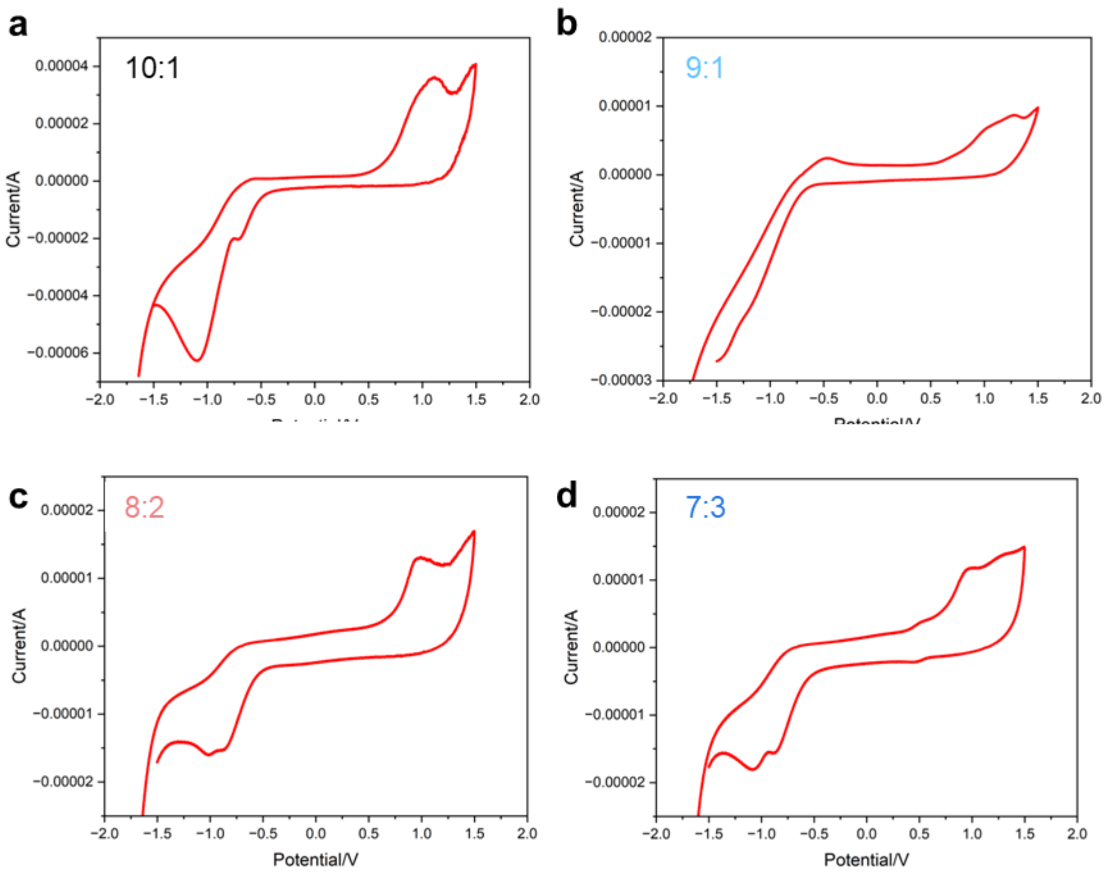

Figure S28. Cyclic voltammetry of PBTTT with MCM moiety.

MCM ratio	HOMO [a] (eV)	LUMO [b] (eV)	E_g^{opt} (eV)
10:0	-5.03	-2.84	2.19
9:1	-5.10	-2.85	2.25
8:2	-5.11	-2.88	2.23
7:3	-5.12	-2.9	2.22

Table S2. Electrochemical properties of PBTTT with MCM moiety. [a] The frontier energy levels calculated from the cyclic voltammetry; [b] LUMO = HOMO – E_g^{opt}.

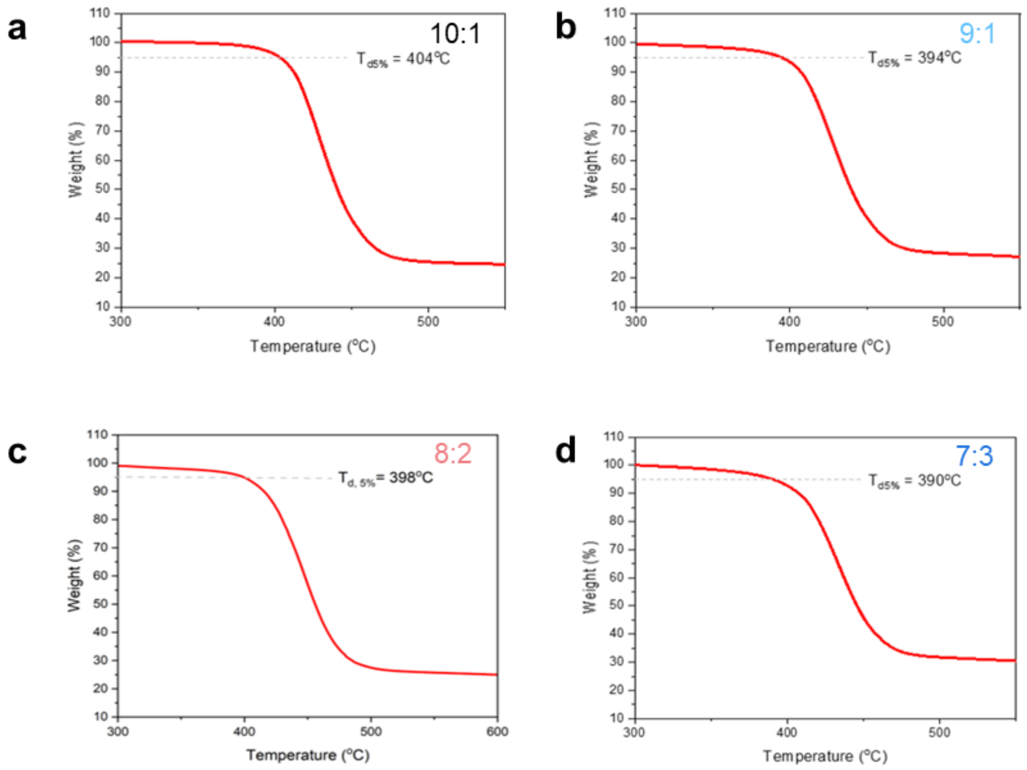

Figure S29. Thermogravimetric analysis (TGA) of PBTTT with MCM moiety

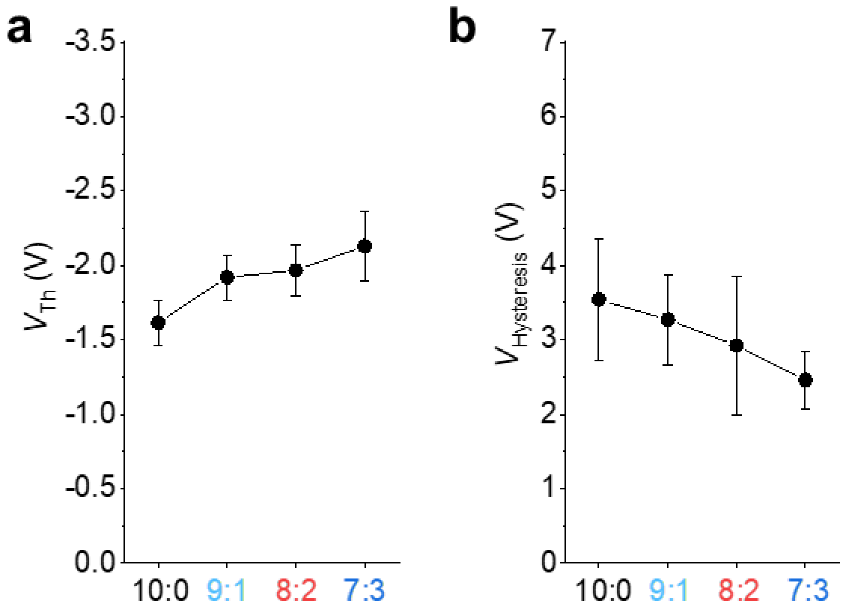

Figure S30. Threshold voltage and hysteresis in transfer curves according to MCM ratio.

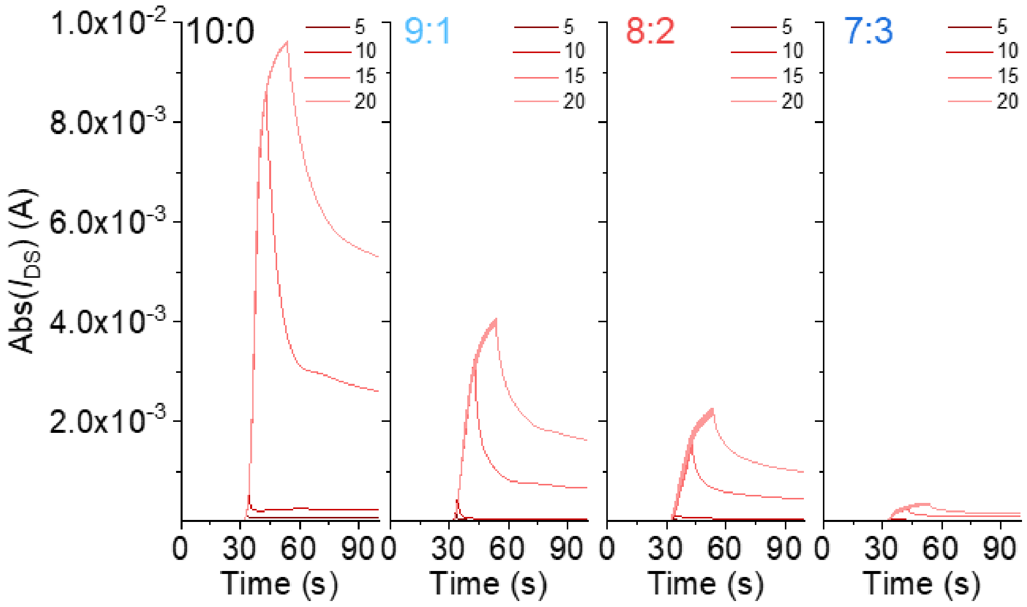

Figure S31. Spike responses according to MCM ratio.

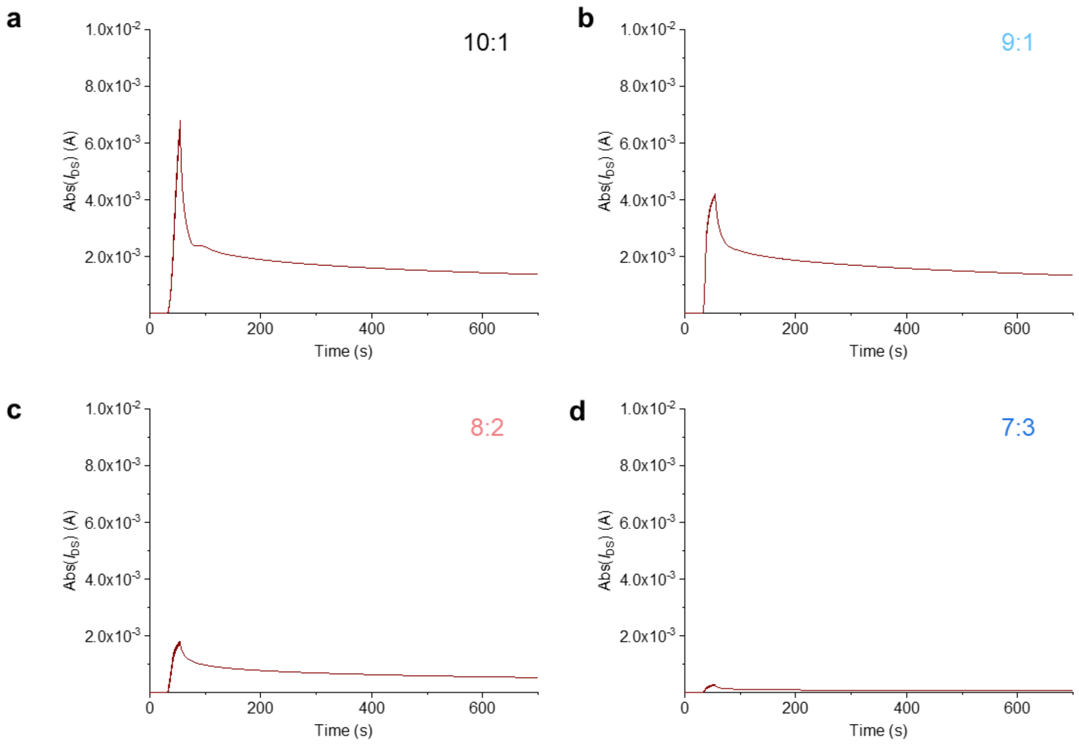

Figure S32. Decay current measurements after spike response.

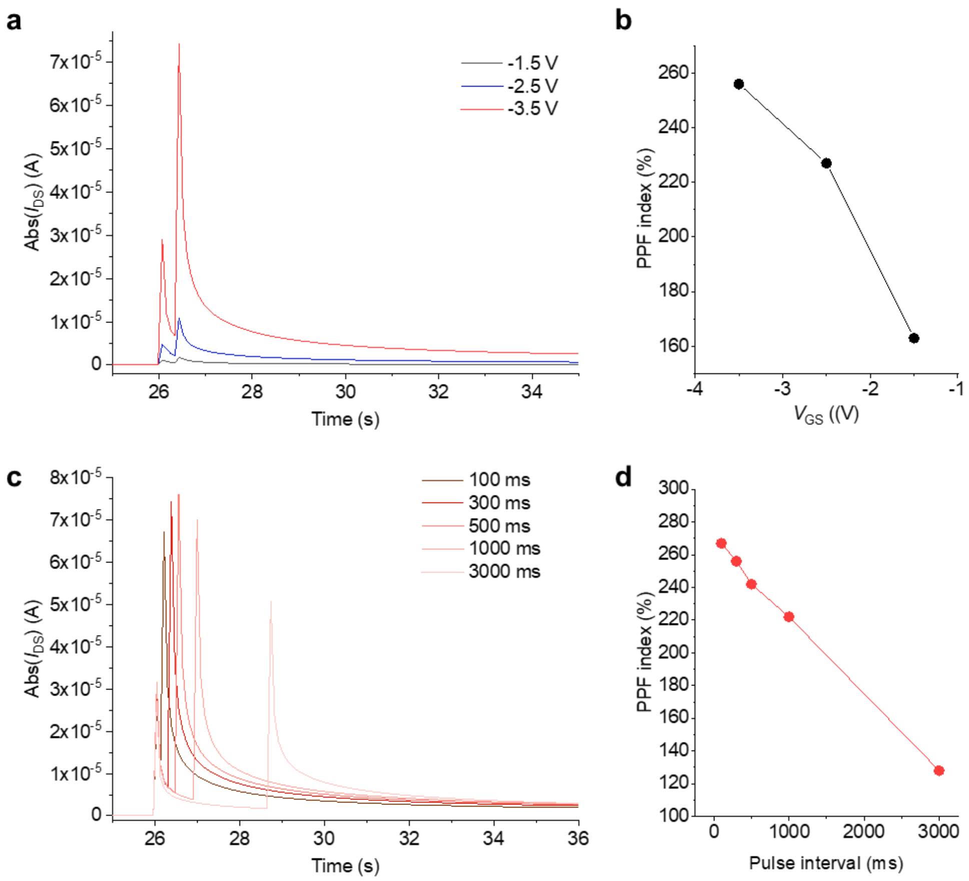

Figure S33. SVDP and SRDP of MCM ratio 8:2 polymer.

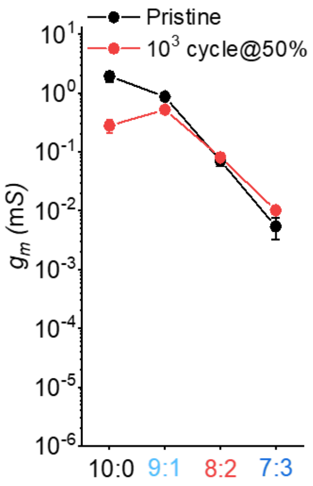

Figure S34. Transconductance (g_m, mS) values and relative changes after cyclic stretching.

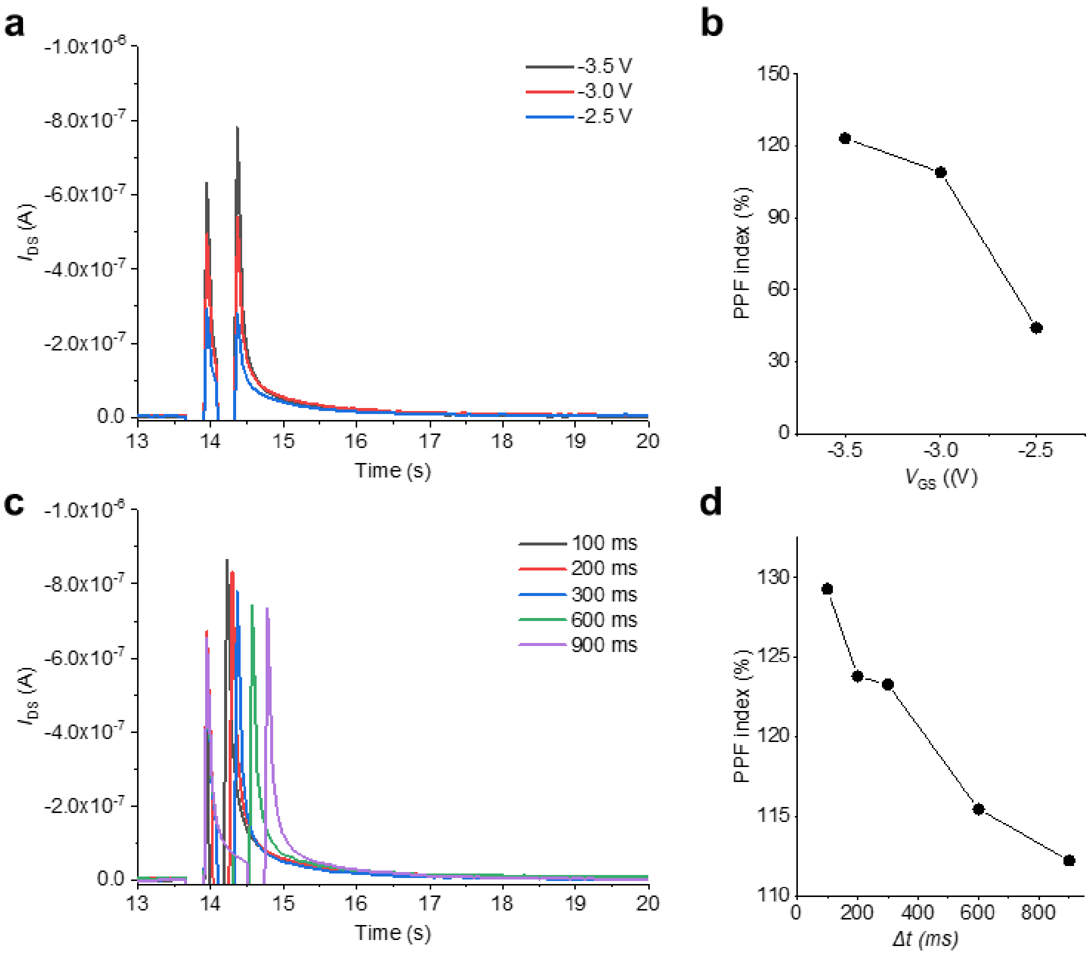

Figure S35. SVDP and SRDP of ISND using MCM ratio 8:2 polymer.

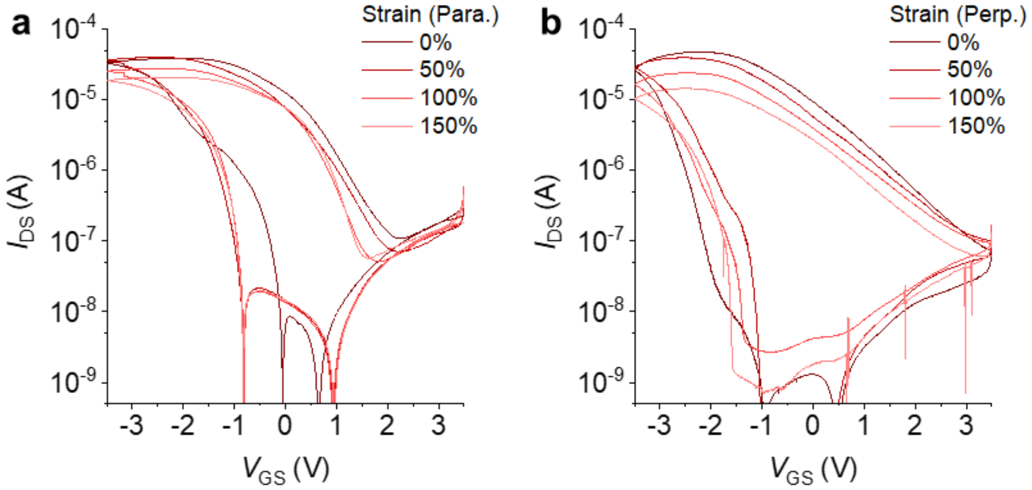

Figure S36. Transfer curves of ISND with MCM 8:2 polymer under strain**.**

Table S3. **Comparison of mechanical and electrical performance in transfer curves of ISNDs incorporating organic semiconductors and solid gel electrolytes**

Semiconductor	MAX static strain	Retained current	MAX cycle	Retained current	References
P3HT/PDMS	50%	Perp. ~ 25%	-	-	Sci. Adv. 5, eaax4961 (2019)
P3HT/PDMS	30%	Perp. ~ 35%<br>Para. ~ 49%	-	-	Nano Res. 15, 2, 758–764 (2022)
p(gT2)	100%	Para. ~48%<br>Perp. ~62%	-	-	Matter 5, 3375–3390 (2022)
PEDOT:PSS/Glycerol	30%	Para. ~21%<br>Perp. ~23%	15%, 100 cycles	Para. ~51%<br>Perp. ~68%	ACS Appl. Mater. Interfaces 15, 41656−41665 (2023)
PEDOT:PSS/PAAMPSA	40%	55%	-	-	InfoMat. 5, 2, e12472 (2023)
DPPDTSE/SEBS	50%	Para. ~33%<br>Perp. ~38%	-	-	Science 380, 735–742 (2023)
P3HT/PDMS	50%	Para. ~67%<br>Perp. ~86%	30%, 500 cycles	Para. ~87%<br>Perp. ~101%	Nano Res. 17, 6550–6559 (2024)
P3HT/SEBS	30%	81%	-	-	Current Opinion in Solid State & Materials Science, 29, 101142 (2024)
PEDOT:PSS/glycerol /GOPS/PEGDE/DBSA	50%	Para. ~91%<br>Perp. ~93%	-	-	Nat. Electron 7, 1176–1185 (2024)
p(gT2)	100%	-	50%, 1000 cycles	~50%-	ACS Materials Lett. 7, 4, 1394–1399 (2025)
PTDPPSe-6Si	50%	Para. ~43%<br>Perp. ~51%	50%, 500 cycles	Para. ~63%	npj Flex Electron 9, 16 (2025)
PEDOT:PSS/Triton X	30%	Para. ~41%<br>Perp. ~52%	30%, 1000 cycles	Para. ~21%	Nano Research (2025) https://doi.org/10.26599/NR.2025.94907780
PBTTT -MCM 8:2	150%	**(50%)<br>Perp. ~82%<br>Para. ~102%<br>(150%)<br>Perp. ~54%<br>Para. ~30%**	**-**	**-**	This work

Table S4. **Comparison of mechanical and electrical performance in spike responses of ISNDs incorporating organic semiconductors and solid gel electrolytes**

Semiconductor	MAX static strain	Retained current	MAX cycle	Retained current	References
P3HT/PDMS	50%	60%	-	-	Sci. Adv. 5, eaax4961 (2019)
P3HT/PDMS	30%	54%	-	-	Nano Res. 15, 2, 758–764 (2022)
p(gT2)	100%	66%	100%, 100 cycles	81%	Matter 5, 3375–3390 (2022)
PEDOT:PSS/Glycerol	-	-	-	-	ACS Appl. Mater. Interfaces 15, 41656–41665 (2023)
PEDOT:PSS/PAAMPSA	40%	-	-	-	InfoMat. 5, 2, e12472 (2023)
DPPDTSE/SEBS	-	-	-	-	Science 380, 735–742 (2023)
P3HT/PDMS	-	-	-	-	Nano Res. 17, 6550–6559 (2024)
P3HT/SEBS	30%	77%	-	-	Current Opinion in Solid State & Materials Science, 29, 101142 (2024)
PEDOT:PSS/glycerol /GOPS/PEGDE/ DBSA	-	-	-	-	Nat. Electron 7, 1176–1185 (2024)
p(gT2)	-	-	-	-	ACS Materials Lett. 7, 4, 1394–1399 (2025)
PTDPPSe-6Si	50%	61%	-	-	npj Flex Electron 9, 16 (2025)
PEDOT:PSS/Triton X	30%	-	-	-	Nano Research (2025) https://doi.org/10.26599/NR.2025.94907780
PBTTT -MCM 8:2	**50%**	**101%**	**10^5**	**98%**	This work

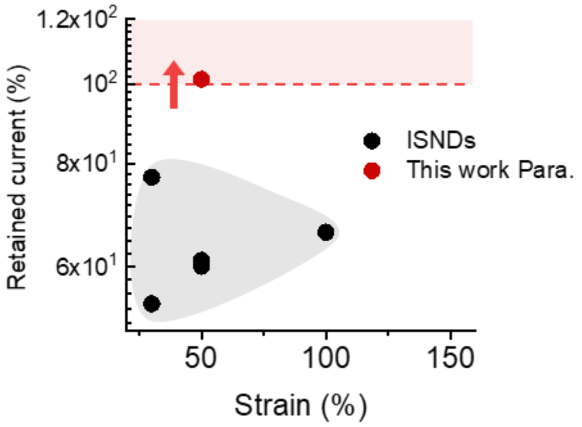

Figure S37. Comparison plot of reported stretchability in spike response of ISNDs

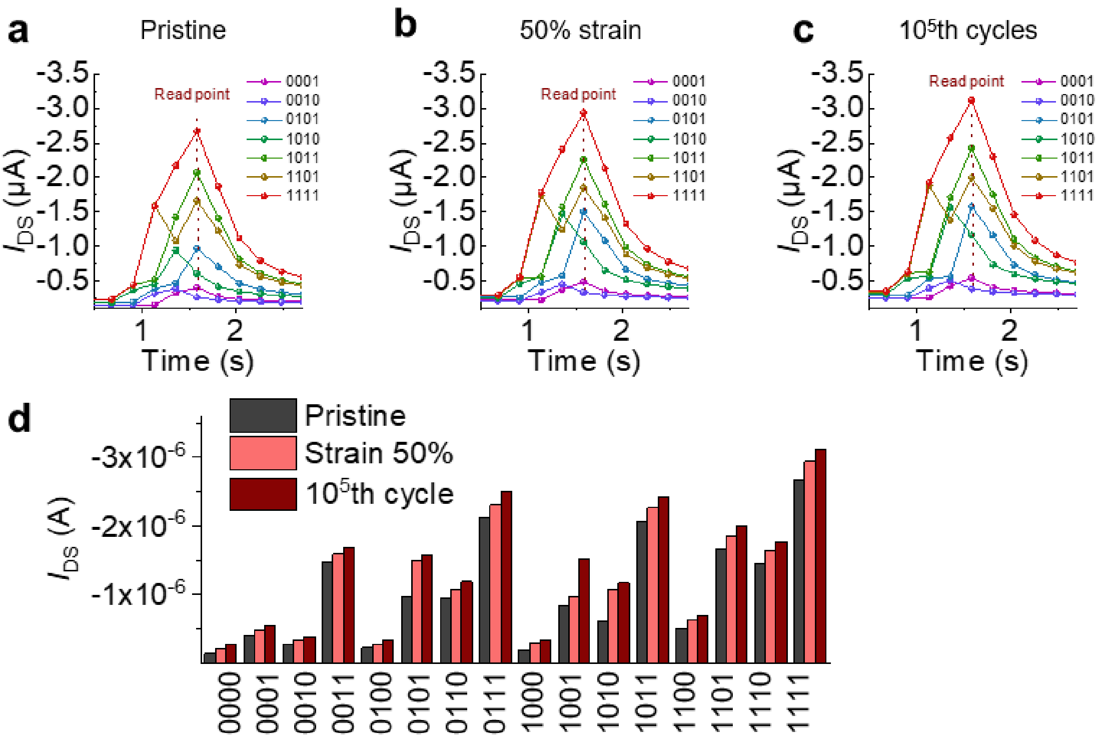

Figure S38. Spike responses and multistate of ISND physical reservoir**.**

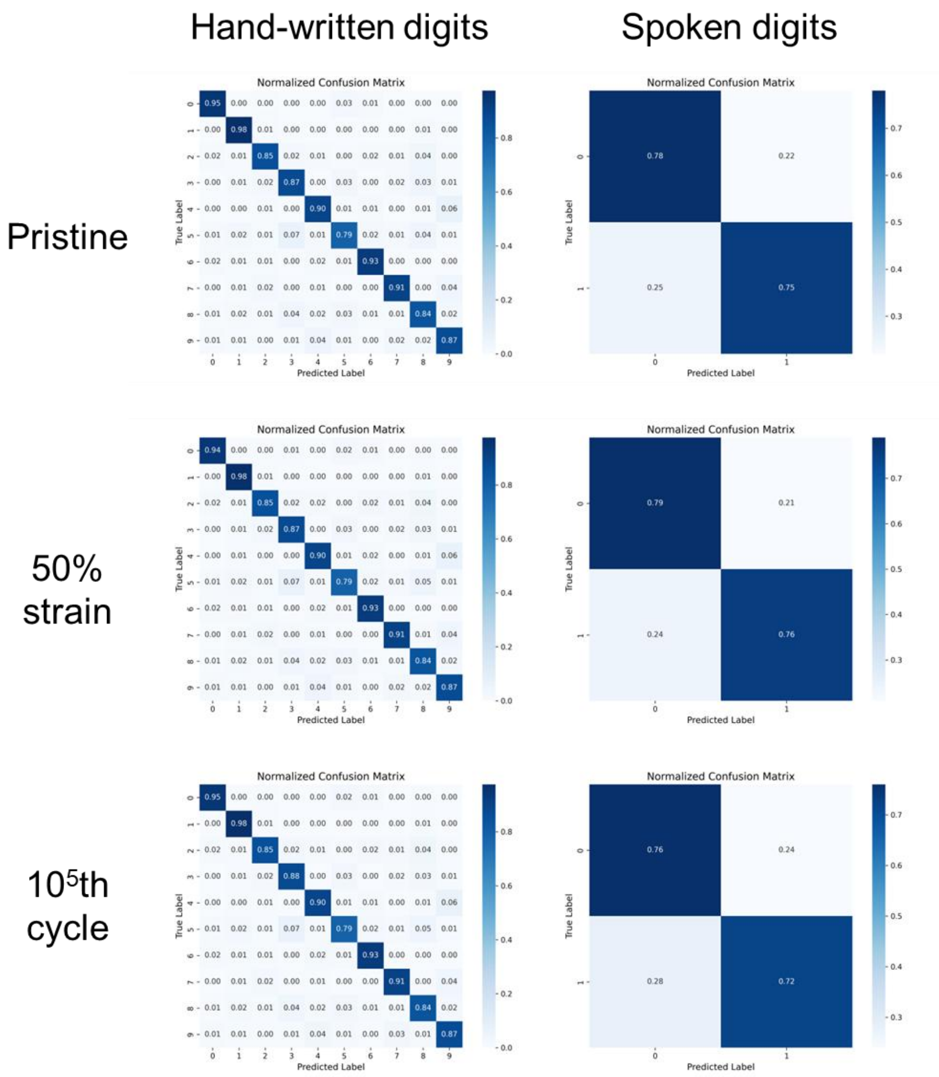

Figure S39. Color confusion matrix plots

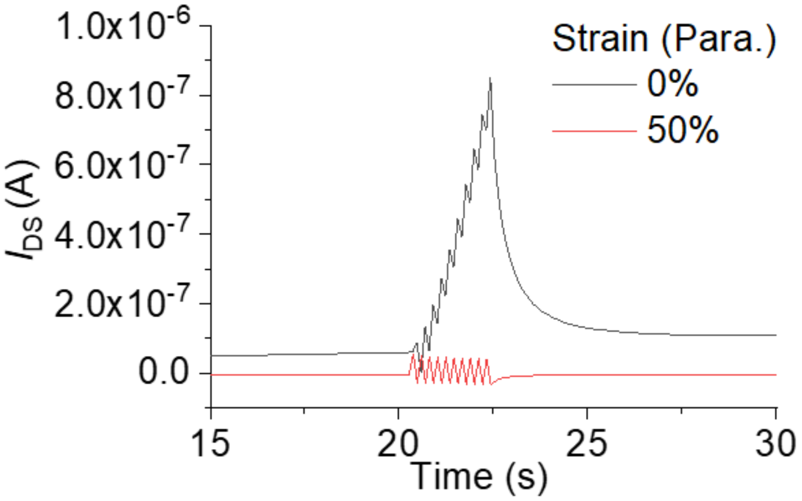

Figure S40. Spike response of ISND using 10:0.
